# *Cis*‐ and *Trans*‐Regulatory Factors Independently Shape Phenotypic Heterogeneity of Retinitis Pigmentosa

**DOI:** 10.1002/advs.202520828

**Published:** 2026-04-10

**Authors:** Cong Cui, Kotone Nakagawa, Takumi Tateno, Ayaka Dan, Dexin Meng, Yoshihiro Omori, Soma Tomihara, Suzuri Okamoto, Shigeru Sato, Motokazu Tsujikawa

**Affiliations:** ^1^ Laboratory of Regenerative Medicine and Development Graduate School of Medicine The University of Osaka Osaka Japan; ^2^ Department of Ophthalmology The Second Affiliated Hospital of Harbin Medical University Harbin Medical University Harbin China; ^3^ Graduate School of Integrated Sciences for Life Hiroshima University Hiroshima Japan; ^4^ Reverse Translational Research Project National Institutes of Biomedical Innovation Health and Nutrition (NIBN) Osaka Japan

**Keywords:** cis‐regulatory variant, phenotypic heterogeneity, retinitis pigmentosa, trans‐acting genetic modifier

## Abstract

Retinitis pigmentosa (RP) is a group of retinal inherited diseases characterized by substantial clinical heterogeneity, even among individuals carrying the same pathogenic mutation. In this study, we identified two distinct genetic mechanisms influencing RP severity using a transgenic zebrafish model expressing the human rhodopsin S334X mutation. The first mechanism is tightly linked to RP onset and results in a benign phenotype. Whole‐genome sequencing revealed a 3‐base‐pair insertion upstream of the transgene, specific to the benign line, acting as a *cis*‐regulatory variant that suppresses transgene expression, as validated by RT‐qPCR, Western blot and luciferase assays. The second mechanism operates independently of RP onset and modifies the benign phenotype toward a more severe presentation. This segregation was observed in approximately half of the offspring derived from specific individuals within the S334X‐benign line, consistent with a Mendelian ratio, suggesting a dominant *trans*‐acting factor. Together, these findings provide the first in vivo evidence that both *cis*‐ and *trans*‐regulatory elements independently and cooperatively modulate RP phenotypes within a shared genetic background. They clearly establish the existence of genetic modifiers as key contributors to disease variability, offering new directions for personalized RP management.

## Graphical abstract

A zebrafish model carrying an identical human *RHO* S334X allele reveals two independent genetic layers shaping retinitis pigmentosa (RP) severity: a protective 3‐bp cis‐regulatory insertion that attenuates transgene expression, and a dominant trans‐acting modifier that restores a severe phenotype. This dual‐layer architecture provides direct in vivo evidence for modifier‐driven phenotypic heterogeneity in RP.

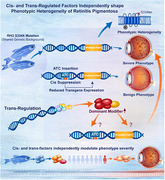

## 1 Introduction

Retinitis pigmentosa (RP) comprises a genetically heterogeneous group of retinal diseases marked by progressive photoreceptor cell loss, ultimately resulting in vision impairment or blindness [1, 2]. A hallmark of RP is its pronounced heterogeneity, which manifests at both the genetic and clinical levels [3]. One major form of genetic heterogeneity is locus heterogeneity, wherein mutations in different genes can lead to similar clinical phenotypes. To date, more than 300 disease‐causing genes have been mapped, encompassing a broad spectrum of inheritance patterns, including autosomal dominant, autosomal recessive, X‐linked, digenic, and mitochondrial inheritance [4, 5]. This extensive locus heterogeneity poses significant challenges for genetic diagnosis, as individuals with indistinguishable clinical presentations may harbor mutations in entirely different genes [6, 7].

In addition to its genetic complexity, RP exhibits substantial clinical heterogeneity [1, 8, 9]. Even individuals carrying the same pathogenic mutation at a single genetic locus can exhibit markedly different disease trajectories. For instance, siblings carrying the same pathogenic mutation may exhibit contrasting visual outcomes, ranging from early onset, rapidly progressing retinal degeneration to mild symptoms with preserved vision into adulthood [10]. Such variability complicates prognosis and counseling, implicating modifiers beyond the primary mutation [9, 11].

Both external and genetic factors have been proposed to contribute to the clinical variability observed in RP [12, 13]. In several Mendelian disorders, genetic background effects are well established, and reproducibly validated genetic modifiers—such as SMN2 copy number in spinal muscular atrophy and the HbF‐regulatory loci BCL11A and HBS1L–MYB in sickle cell disease—demonstrate that modifier genes can exert substantial influence on disease severity [14, 15]. However, in inherited retinal diseases (IRDs), including RP, robustly validated genetic modifiers remain comparatively limited.

Among external factors, oxidative stress and light exposure have been extensively studied in RP models, with numerous investigations demonstrating their significant impact on disease progression [16]. Light‐induced retinal degeneration activates multiple apoptotic pathways [17], and is exacerbated by oxidative damage in models with compromised antioxidant defenses [18]. Studies—including our own [12, 19]–have shown that even low‐intensity light exposure can accelerate retinal degeneration.

Despite these insights, identifying genetic modifiers that contribute to RP clinical heterogeneity remains a major challenge. We identified a zebrafish model that, despite carrying a uniform pathogenic allele at a single genetic locus, exhibited genetically driven phenotypic divergence. C‐terminal truncating mutations of rhodopsin, such as S334X and Q344X, are well‐characterized examples that disrupt the VxPx ciliary targeting signal, leading to opsin mislocalization and early‐onset photoreceptor degeneration in vertebrate systems. Here, using a zebrafish model carrying an identical pathogenic *RHO* S334X allele, we investigate how genetic factors beyond the primary mutation modulate disease severity.

## 2 Results

### 2.1 Establishment of a *Rhodopsin* S334X Zebrafish Model

*Rhodopsin* mutations account for approximately 10% of all RP cases and represent the most common cause of autosomal dominant RP [7]. Among these, C‑terminal truncating mutations that abolish the conserved VxPx ciliary‑targeting signal constitute a functional hotspot associated with the most severe, early‑onset phenotypes and are classified as Class I rhodopsin mutations [20, 21].  Loss of the VxPx motif disrupts ciliary trafficking, leading to opsin mislocalization and rapid photoreceptor degeneration in vertebrate models [20, 21, 22]. This framework provides a strong biological rationale for using VxPx‐truncating alleles such as Q344X and S334X as robust RP models in the present study.

Over the past decade, our laboratory has developed zebrafish models of RP by expressing human mutant *rhodopsin* under the control of the zebrafish *rhodopsin* (*rh1*) promoter [19, 23]. Among these, the Q344X transgenic line has been established as a robust RP model [19, 24], whereas the S334X transgenic line has been suggested to act as a robust RP model [25].

We therefore examined rod photoreceptor numbers specifically in the S334X transgenic fish and their wild‑type (WT) siblings at 5 and 7 days post‑fertilization (dpf).

**FIGURE 1:**
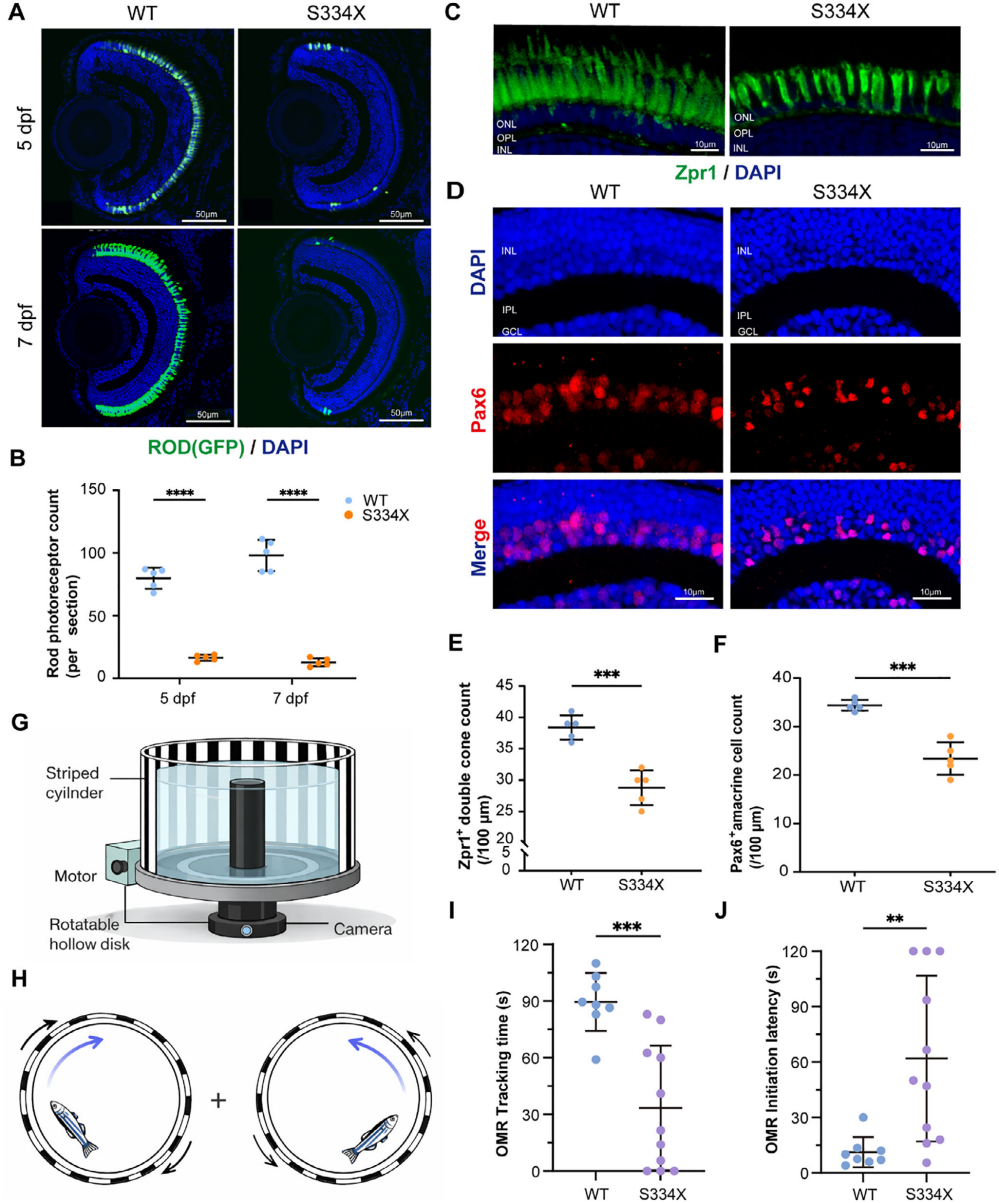
Phenotypes of the Tg (rh1:hs*RHO*;Omp:EGFP) Transgenic Zebrafish Model. (A) Retinal cryosections from RHO:S334X and WT zebrafish at 5 and 7 dpf. Rod photoreceptors are visualized by *rh1*:EGFP (green), and nuclei are counterstained with DAPI (blue). Scale bars: 50 µm (upper panels). (B) Quantification of rod photoreceptor numbers at 5 and 7 dpf. RHO:S334X zebrafish show significantly reduced rod cell counts compared with WT at both time points (****P < 0.0001, unpaired two‐tailed Student's t‐test; error bars represent SD; *n* = 5 per group). (C) Representative retinal cryosections from 15 dpf WT and S334X zebrafish stained with Zpr1 (green) and DAPI (blue). Scale bar: 10 µm. (D) Representative retinal cryosections from 15 dpf zebrafish stained for Pax6 (red) and DAPI (blue). Pax6^+^ cells are predominantly localized in the inner nuclear layer (INL), with occasional expression observed in the ganglion cell layer (GCL). INL, inner nuclear layer; IPL, inner plexiform layer; GCL, ganglion cell layer. Scale bar: 10 µm. (E) Quantification of Zpr1^+^ cells shown as the number of double cones per 100 µm of retinal length. (*n* = 5 per group). Statistical significance was assessed using an unpaired two‐tailed Student's t‐test (***P < 0.001). (F) Quantification of amacrine cells, shown as the number of Pax6‐positive cells in the INL per 100 µm of retinal length (*n* = 5 per group). Statistical significance was assessed using an unpaired two‐tailed Student's t‐test (***P < 0.001). (G) Schematic illustration of the optomotor response (OMR) assay setup. Zebrafish were placed in a transparent cylindrical arena surrounded by a vertically striped rotating cylinder driven by a motor. A rotatable hollow disk was positioned beneath the arena, and zebrafish swimming behavior was recorded from below using a camera. (H) Experimental design of the OMR assay. Juvenile zebrafish (40 dpf) were sequentially exposed to clockwise and counterclockwise rotation of the striped pattern for 2 min per direction, and swimming behavior was recorded from below. (I, J) Quantification of tracking time (I) and initiation latency (J) during the optomotor response (OMR) assay in juvenile zebrafish (40 dpf). Tracking time was defined as the total duration spent swimming in the direction of the rotating striped pattern, whereas initiation latency was defined as the time required to initiate directional swimming following stimulus onset. WT fish exhibited longer tracking times and shorter initiation latency compared with the S334X‐original line (WT, *n* = 8; S334X‐original line, *n* = 11). Statistical significance was assessed using a two‐tailed Mann–Whitney U test (I, ***P < 0.001; J, **P < 0.01). Error bars represent SD.

At both time points, zebrafish expressing the S334X mutation exhibited significantly reduced rod photoreceptor counts compared with WT controls. (5 dpf: S334X: 16.4 ± 2.41 cells/section, WT: 79.8 ± 8.38 cells/section, *n* = 5 per group, *P* = 2.06 × 10^−^^7^; 7 dpf: S334X: 12.8 ± 3.19 cells/section, WT: 98.0 ± 12.41 cells/section, *n* = 5 per group, *P* = 4.13 × 10^−^^7^) (Figure 1A,B).

We next quantified cone photoreceptors and amacrine cells at 15 dpf to assess whether additional retinal cell types were affected in the S334X line. Both populations were significantly reduced in S334X fish compared with WT (Figure 1C–F) (double cone: WT: 38.4 ± 1.95, S334X: 28.8 ± 2.78, *n* = 5 per group; *P* = 0.0002; amacrine: WT: 34.4 ± 1.14, S334X: 23.4 ± 3.36, *n* = 5 per group; *P* = 0.0001); however, the magnitude of reduction was milder than that observed for rods. These findings indicate that degeneration in the S334X line is predominantly rod‐initiated, with later involvement of non‐rod neurons. This sequence mirrors the clinical course of RP and is consistent with patterns reported for the Q344X model, supporting the suitability of S334X as an RP model.

Given that structural analyses indicated rod‐predominant degeneration in the S334X line, we next examined whether these anatomical deficits translated into measurable functional impairment using the optomotor response (OMR) assay [26]. OMR is a visually guided behavioral paradigm widely used to evaluate visual function in zebrafish. In the OMR assay, fish are placed in a cylindrical tank whose inner wall is surrounded by rotating black‐and‐white stripes, while the tank itself remains stationary and no water current is generated. Visually intact fish reflexively swim in the same direction as the rotating pattern (“tracking”), whereas fish with impaired vision exhibit reduced tracking time and delayed initiation of tracking (i.e., prolonged latency) [26] (Figure 1G,H).

S334X‐original zebrafish exhibited a marked reduction in OMR performance, characterized by significantly shortened tracking time and a pronounced delay in response initiation (Figure 1I, J; Video S1 (Wildtype), Video S2 (S334X)) (tracking time: WT: 89.56 ± 15.35, *n* = 8; S334X‐original line: 33.41 ± 33.00, *n* = 11; *P* = 0.0003; Initiation latency: WT: 11.25 ± 8.20; S334X‐original line: 61.91 ± 44.86; *P* = 0.0036).

Together, these structural and functional analyses demonstrate that the S334X zebrafish is an appropriate and reliable model of RP.

### 2.2 Identification of a Benign Subset in the S334X Line Exhibiting Attenuated Retinal Degeneration

**FIGURE 2:**
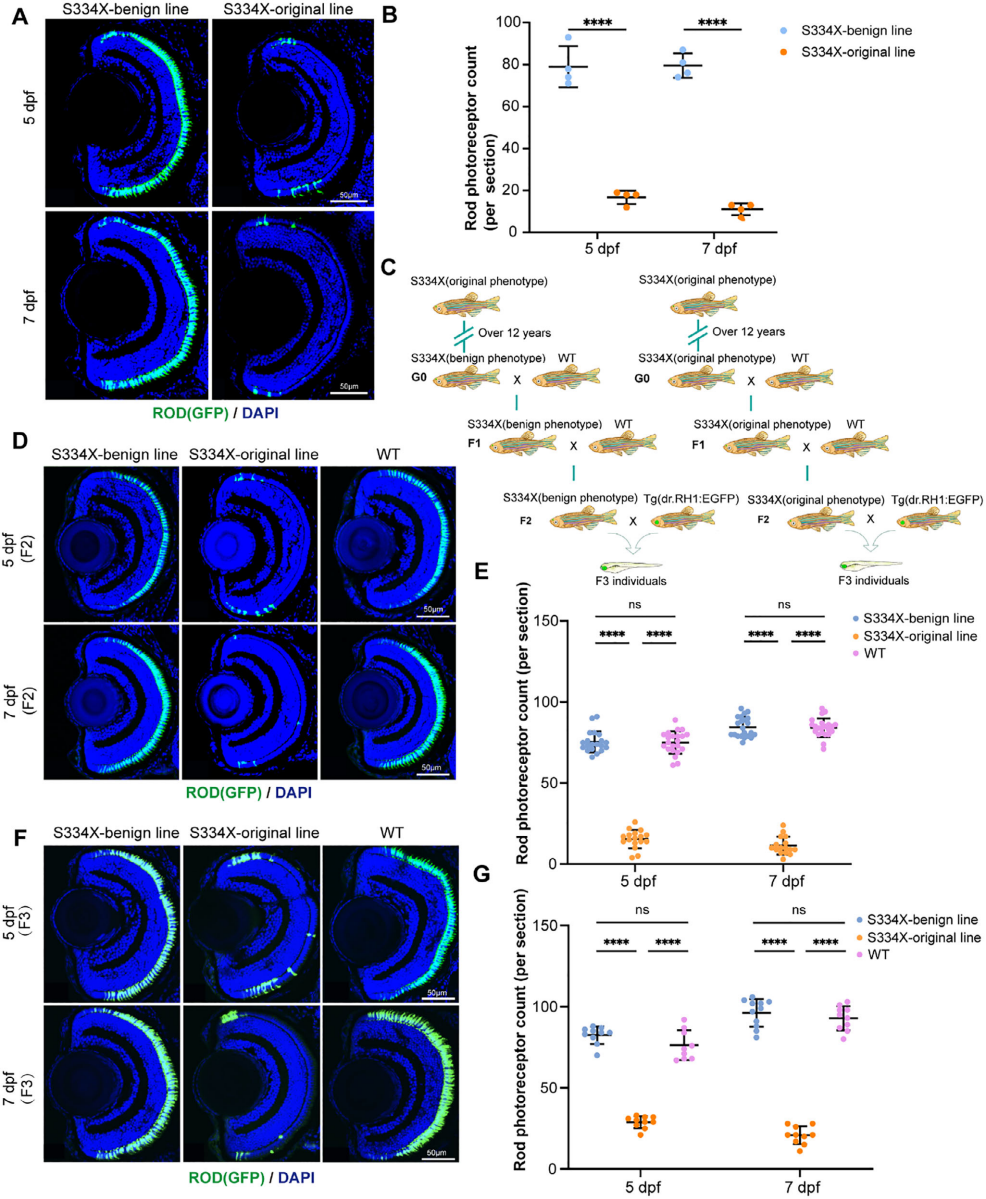
Identification of a Genetically Distinct Phenotypic Line in *RHO*:S334X Zebrafish. (A) Retinal cryosections from S334X‐benign and S334X‐original zebrafish at 5 and 7 dpf. Rod photoreceptors are labeled with EGFP (green), and nuclei are counterstained with DAPI (blue). Scale bar: 50 µm. (B) Quantification of rod photoreceptor numbers at 5 and 7 dpf, showing significantly higher rod cell counts in the S334X‐benign line compared with the S334X‐original line at both time points (****P < 0.0001, unpaired two‐tailed t‐test; error bars represent SD; *n* = 4 per group). (C) Schematic overview of the breeding strategy. The original S334X transgenic line exhibited a severe phenotype. After more than 12 years of breeding, a benign variant was identified and designated as a distinct line. Both the original and benign S334X lines were independently crossed with *rh1*:EGFP transgenic fish to visualize rod photoreceptors. F1 progeny were propagated to establish stable F2 generations, and individual F2 fish were further outcrossed to generate F3 progeny for phenotypic analysis. (D) Retinal cryosections from WT, S334X‐benign, and S334X‐original zebrafish in the F2 generation at 5 and 7 dpf. Rod photoreceptors are labeled with EGFP (green), and nuclei are counterstained with DAPI (blue). Scale bar: 50 µm. (E) Quantification of rod photoreceptors in F2 zebrafish (one‐way ANOVA followed by Tukey's multiple comparisons test; ****P < 0.0001; ns, not significant; error bars represent SD; *n* = 16–22 per group). (F) Retinal cryosections from WT, S334X‐benign, and S334X‐original zebrafish in the F3 generation at 5 and 7 dpf, showing stable inheritance of the benign phenotype. Scale bar: 50 µm. (G) Quantification of rod photoreceptors in F3 zebrafish, demonstrating that rod cell counts in the S334X‐benign line remained significantly higher than in the S334X‐original line and comparable to WT (****P < 0.0001; ns, not significant; error bars represent SD; *n* = 9–11 per group).

The S334X transgenic line has been maintained in our laboratory for over 12 years through routine outcrossing with the AB wild‐type strain. During this period, we unexpectedly identified a single S334X founder whose progeny exhibited a markedly attenuated retinal degeneration phenotype. We termed this new phenotype the “S334X‐benign” line, whereas the previously established phenotype is referred to as the “S334X‐original” line. Histological analysis confirmed that all transgene‐positive offspring from the S334X‐benign founder displayed substantially higher rod photoreceptor numbers than the S334X‐original line at 5 and 7 dpf (Figure 2A,B).  (5 dpf: benign: 79.0 ± 9.76 cells/section, original: 16.75 ± 3.20 cells/section, *n* = 4 per group, *P* = 1.92 × 10^−^^5^; 7 dpf: benign: 79.5 ± 5.80 cells/section, original: 11.0 ± 2.83 cells/section, *n* = 4 per group, *P* = 7.13 × 10^−^^7^).

The F1 generation was further outcrossed with WT fish to produce the F2 generation, allowing us to assess whether the benign phenotype was heritable and genetically stable across generations (Figure 2C). At both 5 and 7 dpf, F2 individuals from the benign line exhibited rod counts that were at least approximately 4‐fold higher than those from the original line (Figure 2D, E) (5 dpf: benign: 75.4 ± 6.49 cells/section, *n* = 20, original: 15.47 ± 5.58 cells/section, *n* = 17, *P* < 1×10^−^^16^; 7 dpf: benign: 84.55 ± 6.19 cells/section, *n* = 22, original: 11.44 ± 5.54 cells/section, *n* = 16, *P* < 1×10^−^^16^). Rod counts in the benign line were statistically indistinguishable from WT controls at both time points (5 dpf: WT: 75.05 ± 6.97 cells/section, *n* = 22, *P* = 0.983; 7 dpf: WT: 84.23 ± 5.81 cells/section, *n* = 22, *P* = 0.982). In contrast, the original line showed significantly reduced rod numbers relative to WT (5 dpf: *P* < 1×10^−^^16^; 7 dpf: *P* < 1×10^−^^16^).

To further confirm the linkage between the benign phenotype and the RP transgene, we examined its inheritance in the F3 generation (Figure 2F). A subset of F2 fish was first verified histologically to ensure the parental benign phenotype, and the remaining siblings were crossed with WT fish to generate the F3 progeny. For comparison, the original S334X line used in this study was independently derived from a G0 founder distinct from the benign lineage. The benign phenotype was stably inherited in the F3 generation, with rod photoreceptor counts significantly higher than those of the original line at both examined stages (Figure 2F,G). Notably, all F3 benign individuals exhibited rod numbers within the physiological WT range, and no severe‐like phenotypes were observed.

In the F3 generation, segregation of hemizygotes carrying the transgene (identified by nasal fluorescence) followed a Mendelian 1:1 ratio relative to their WT siblings (WT: 150; transgenic: 133; *n* = 283, Table 1; and Figure S1). Among the 133 transgenic individuals, 60 fish were randomly selected for histological analysis of rod photoreceptor numbers. All examined fish exhibited the benign phenotype, with the minimum rod count (≥70 cells/section) substantially higher than that of the S334X‐original line. Collectively, these observations across three generations demonstrate that the S334X‐benign phenotype is genetically linked to the RP transgene and consistently cosegregates with elevated rod survival.

**TABLE 1:** Observed counts of offspring genotypes(S334X‐benign).

Genotype	Observed count	Expected count	Chi‐square contribution
WT	150	141.5	0.510
RHO: S334X‐benign	133	141.5	0.510
**Total**	283	283	
**Chi‐square (df = 1)**			χ^2^ = 1.02
**P‐value**			P = 0.312

Genotype counts of offspring derived from heterozygous RHO:S334X‐benign × WT zebrafish crosses at 5 dpf. Transgenic individuals were identified by nasal fluorescence. A chi‐square goodness‐of‐fit test was performed to evaluate whether the observed distribution (WT: 150, RHO: S334X‐benign: 133) deviated significantly from the expected Mendelian 1:1 ratio. The resulting chi‐square value was χ^2^ = 1.02 with 1 degree of freedom (df = 1), corresponding to a P value of 0.312. No significant deviation from Mendelian inheritance was detected.

### 2.3 Non‐Rod Retinal Phenotypes and in vivo Visual Function in the S334X‐Benign Line

**FIGURE 3:**
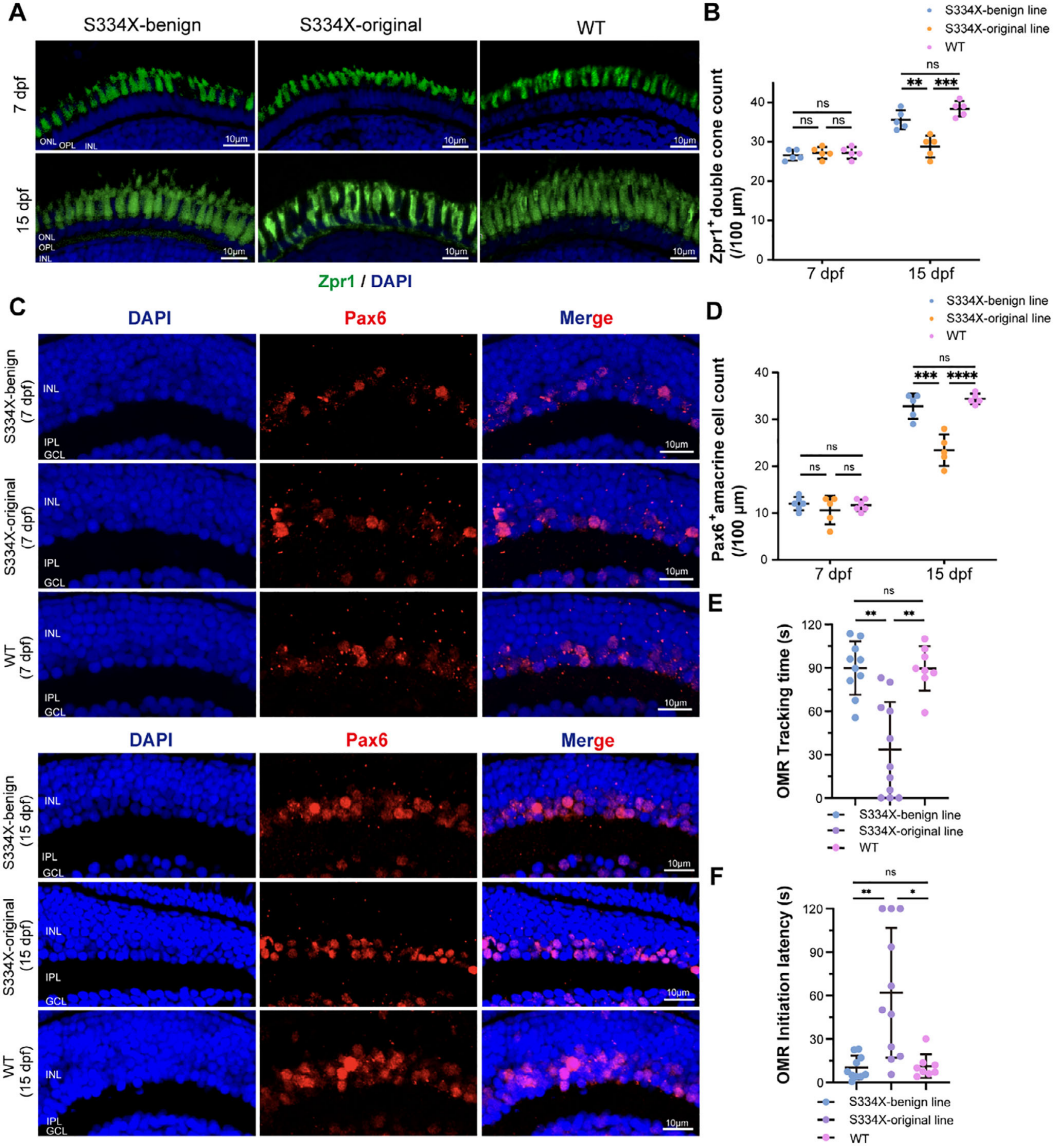
Structural Preservation and Functional Divergence Between Benign and Original RP(S334X) Lines. (A) Representative retinal cryosections from 7 dpf and 15 dpf, WT, S334X‐benign line, and S334X‐original line, stained with Zpr1 (green) and DAPI (blue). Scale bar: 10 µm. (B) Quantification of Zpr1^+^ cells shown as the number of double cones per 100 µm of retinal length (*n* = 5 per group). One‐way ANOVA followed by Tukey's multiple comparisons test (***P < 0.001; **P < 0.01; ns, not significant). (C) Representative retinal cryosections from 7 dpf and 15 dpf zebrafish stained for Pax6 (red) and DAPI (blue). Pax6^+^ cells are predominantly localized in the inner nuclear layer (INL), with occasional expression observed in the ganglion cell layer (GCL). INL, inner nuclear layer; IPL, inner plexiform layer; GCL, ganglion cell layer. Scale bar: 10 µm. (D) Quantification of Pax6^+^ cells in the INL, shown as the number of amacrine cells per 100 µm of retinal length (*n* = 5–6 per group). One‐way ANOVA followed by Tukey's multiple comparisons test (****P < 0.0001; ***P < 0.001; ns, not significant). (E,F) Quantification of tracking time (E) and initiation latency (F) during the optomotor response (OMR) assay in juvenile zebrafish (40 dpf). (*n* = 8–11). Statistical significance was assessed using a Kruskal–Wallis test followed by Dunn's multiple comparisons test (**P < 0.01; *P < 0.05; ns, not significant). Error bars represent SD.

To determine whether phenotypic divergence between the two S334X lines is restricted to rods or extends to other retinal neuronal populations, we quantified cone photoreceptors and amacrine cells and examined the organization of the inner plexiform layer (IPL) at defined stages. At 7 dpf, cone and amacrine cell numbers were indistinguishable between the two lines and comparable to WT; IPL organization also showed no evident differences, indicating rod‐selective divergence at RP onset (Figure 3A–D; Figure S2). By 15 dpf, both cones and amacrine cells were significantly reduced in the S334X‐original line relative to WT, whereas both populations remained indistinguishable from WT in the S334X‐benign line (Figure 3A–D) (double cone: benign line: 35.6 ± 2.40, original line: 28.8 ± 2.78, WT: 38.4 ± 1.95, *n* = 5 per group; benign vs original, *P* = 0.0020; original vs WT, *P* = 0.0001; benign vs WT, *P* = 0.20; amacrine: benign line: 32.8 ± 2.68, original line: 23.4 ± 3.36, WT: 34.4 ± 1.14, *n* = 5 per group; benign vs original, *P* = 0.0002; original vs WT, *P* < 0.0001; benign vs WT, *P* = 0.60). Given this structural preservation in S334X‐benign, we next assessed visually guided behavior by the optomotor response (OMR). The S334X‐benign line exhibited significantly longer tracking time and shorter initiation latency than the S334X‐original line and was statistically indistinguishable from WT for both metrics (Figure 3E, F; Video S3 (Benign), Video S4 (Original), and Video S5 (Wildtype)) (tracking time: benign line: 89.9 ± 18.50, original line: 33.41 ± 33.0, WT: 89.56 ± 15.35, *n* = 8–11 per group; benign vs original, *P* = 0.0016; original vs WT, *P* = 0.0037; benign vs WT, *P* >0.9999; Initiation latency: benign line: 10.3 ± 8.16, original line: 61.9 ± 44.86, WT: 11.3 ± 8.20; benign vs original, *P* = 0.0031; original vs WT, P = 0.0213; benign vs WT, P > 0.9999).

Together, these structural and functional findings show that the S334X‐benign line maintains non‐rod retinal integrity and in vivo visual performance, establishing a clear phenotypic distinction from the S334X‐original line.

### 2.4 Time Course of Retinal Degeneration in the S334X‐Benign Line

**FIGURE 4:**
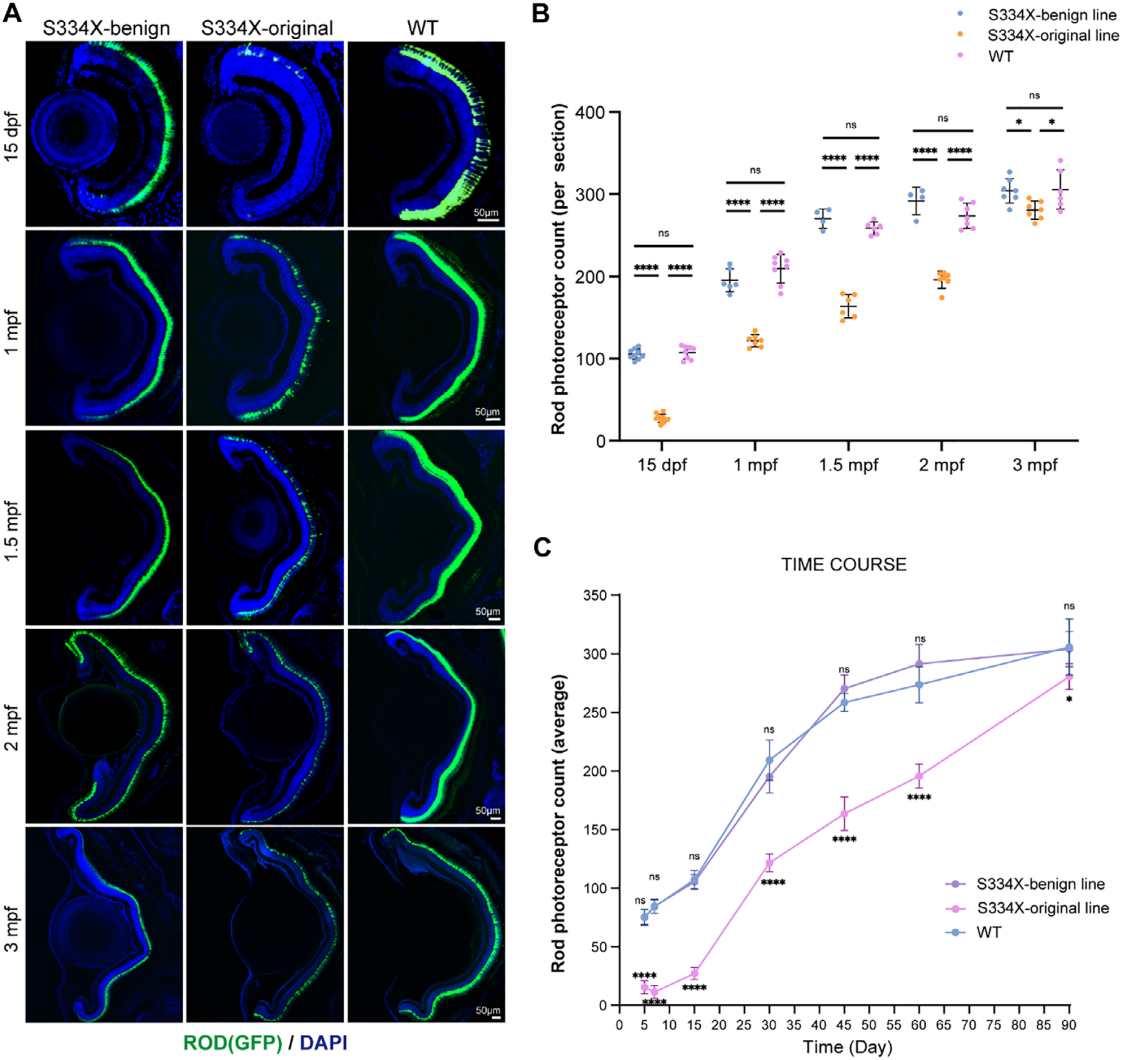
Time‐Course Analysis of Retinal Degeneration in S334X‐benign and S334X‐original Zebrafish. (A) Representative retinal cryosections from S334X‐benign, S334X‐original, and WT zebrafish collected at 15 dpf, 1 mpf, 1.5 mpf, 2 mpf, and 3 mpf. Rod photoreceptors are labeled with EGFP (green), and nuclei are counterstained with DAPI (blue). Scale bar = 50 µm. (B) Quantification of rod photoreceptor numbers from 15 dpf to 3 mpf. The S334X‐benign line consistently maintains significantly higher rod counts than the S334X‐original line at all time points. Rod numbers in the benign group were comparable to WT, whereas the original group showed significantly reduced counts relative to WT and S334X‐benign (one‐way ANOVA followed by Tukey's multiple comparisons test; *P < 0.05, ****P < 0.0001; ns = not significant; error bars represent SD; *n* = 4–10 per group). (C) Time‐course comparison of rod photoreceptor counts between S334X‐benign and S334X‐original zebrafish. Although the gap narrowed by 3 mpf, the difference remained statistically significant, indicating persistent phenotypic divergence over time. Rod counts in the S334X‐benign group remained comparable to WT, while the original group consistently displayed substantial photoreceptor loss. Error bars represent SD.

Given that the S334X‐benign line preserves both retinal structure and visual function at early stages, we next examined whether this attenuated phenotype is maintained throughout the natural course of disease progression. To this end, we monitored rod photoreceptor survival from 15 dpf to 3 months post‐fertilization (mpf) (Figure 4A). At all examined stages, rod photoreceptor numbers in the S334X‐benign line remained markedly higher than those in the S334X‐original line (Figure 4B). At 15 dpf, the benign line exhibited approximately a four‐fold difference relative to the original line (benign: 105.33 ± 6.16 cells/section, original: 27.20 ± 5.05 cells/section, *n* = 9–10, *P* < 1×10^−^^16^), consistent with earlier developmental stages. This pronounced difference persisted at 1, 1.5, and 2 mpf (all *P* < 0.0001). During this period, rod counts in the benign line remained comparable to WT controls, indicating long‐term preservation of near‐physiological rod survival. A summary timecourse plot further illustrates the sustained divergence between the two lines from early development through 3 mpf (Figure 4C). Although the gap between the benign and original lines narrowed by 3 mpf, it remained statistically significant (*P* < 0.05). These findings demonstrate that the S334X‐benign line maintains an attenuated degenerative phenotype over extended developmental periods, in clear contrast to the progressive degeneration observed in the S334X‐original line.

### 2.5 Confirmation of Identical S334X Rhodopsin in Both Lines

**FIGURE 5:**
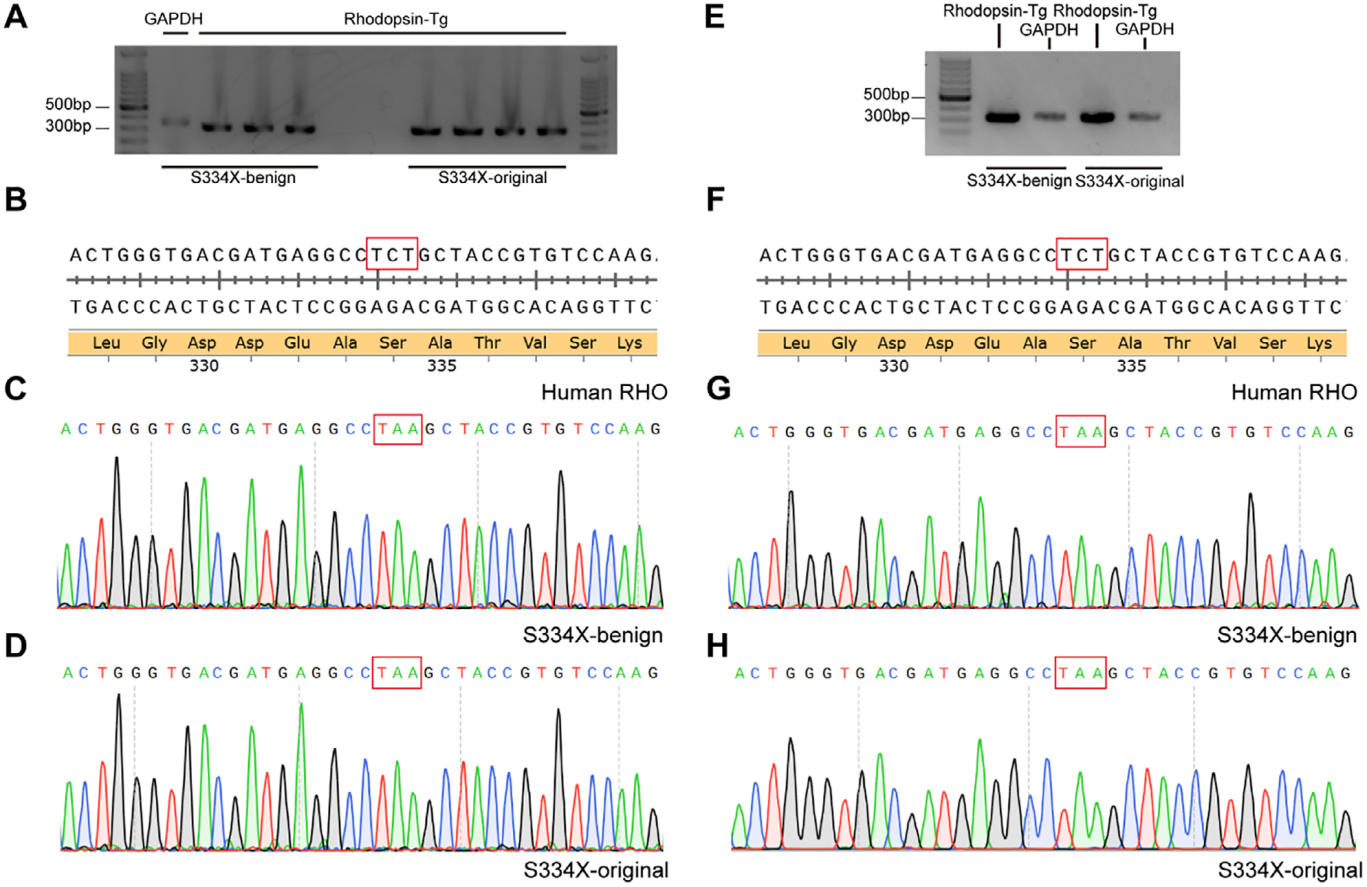
Molecular characterization of the *RHO*:S334X transgene and its expression in S334X zebrafish lines. Validation of the S334X transgene sequence and transcript expression in benign and original zebrafish lines using PCR and Sanger sequencing. (A) Genomic PCR of S334X‐benign (*n* = 3) and S334X‐original (*n* = 4) individuals using primers spanning the human *RHO*:S334X mutation site. The expected 318 bp amplicon was detected in both lines. *GAPDH* was used as an internal control. (B) Reference coding sequence of the human *RHO* gene showing the wild‐type codon 334 (TCT, red box). (C,D) Sanger sequencing of genomic DNA confirms the presence of the TCT → TAA substitution in both S334X‐benign (C) and S334X‐original (D) zebrafish lines. (E) RT‐PCR analysis demonstrating transcript‐level expression of the *RHO*:S334X transgene in both phenotypic lines (*n* = 2 per group). (F) Reference mRNA sequence surrounding codon 334, showing the wild‐type sequence. (G,H) Sanger sequencing of RT‐PCR products confirms transcript‐level expression of the *RHO*: S334X transgene in both phenotypic lines.

Because the benign line exhibited retinal morphology comparable to WT controls, we examined whether sequence changes in the transgene could account for the attenuated phenotype. Genomic DNA from each line (benign, *n* = 3; original, *n* = 4) was subjected to direct sequencing of the integrated human *RHO* transgene. All individuals carried the intended S334X allele—c.1001_1002delCTinsAA, yielding p.Ser334*—and no additional sequence alterations were detected within the human *RHO* coding region in either line (Figure 5A–D). To confirm these findings at the transcript level, cDNA generated from retinally enriched tissue from each line (benign: *n* = 2; original: *n* = 2) was amplified and Sanger sequenced, demonstrating identical transgene transcripts in the two lines (Figure 5E–H).

These results show that both lines possess and express the same S334X mutant human rhodopsin, indicating that the phenotypic divergence is not attributable to sequence variation within the human *RHO* transgene.

### 2.6 Position Effect Ruled Out by Whole‐Genome Sequencing

The S334X line was originally established 12 years ago using a transposon‐based system and has been continuously maintained in our laboratory. However, the genomic insertion site of the transgene was not identified at the time of establishment, raising the possibility that two lines carrying identical transgene constructs might have integrated at distinct genomic loci. In such a scenario, the phenotypic differences we observed could potentially be attributed to position effects—variations in gene expression caused by the genomic context of insertion.

**FIGURE 6:**
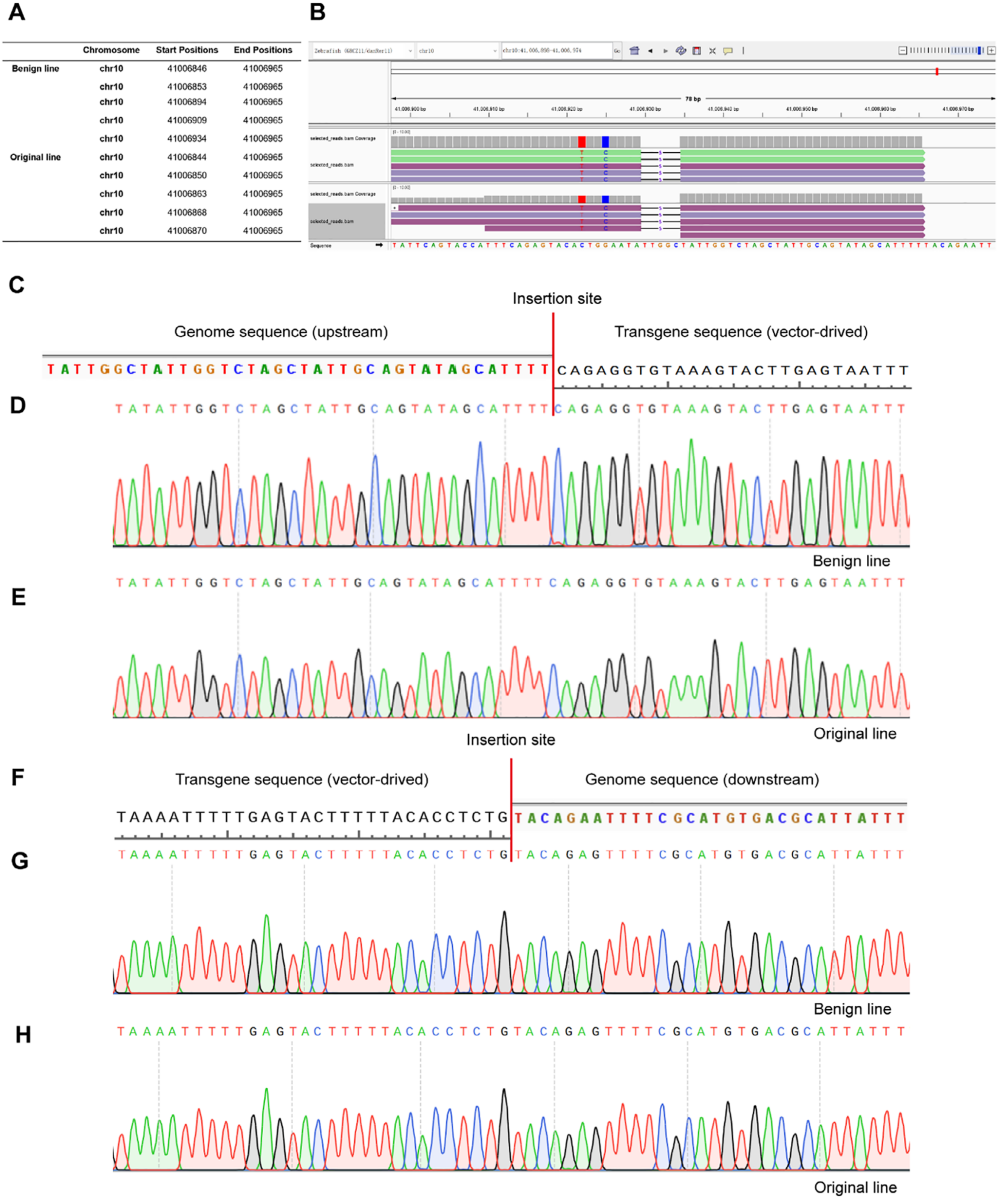
Genomic mapping and validation of transgene insertion in *RHO*:S334X zebrafish lines. Identification of transgene insertion site on chromosome 10 in both benign and original lines using WGS and junction PCR. (A) Whole‐genome sequencing (WGS) identifies an identical transgene insertion site on chromosome 10 at position 41,006,965 in both S334X‐benign and S334X‐original zebrafish lines. (B) Integrated Genome Viewer (IGV) visualization of junction‐spanning reads supports the insertion site in both lines. (C) Schematic illustrating the 5′ genomic integration site of the transgene, showing the junction between the upstream genomic sequence and the 5′ end of the inserted construct on chromosome 10. (D) Sanger sequencing of the 5′ junction confirms integration of the transgene at the genomic site in the S334X‐benign line. (E) Corresponding Sanger sequencing of the 5′ junction in the S334X‐original line shows the same integration site. (F) Schematic diagram illustrating the 3′ genomic integration site of the transgene, depicting the junction between the 3′ end of the inserted construct and the downstream flanking genomic sequence on chromosome 10. (G) Sanger sequencing of the 3′ junction region in the S334X‐benign line confirms seamless integration without detectable rearrangement of the transgene with the genomic sequence at the expected insertion site. (H) Corresponding Sanger sequencing in the S334X‐original line confirms the same 3′ junction structure, consistent with a shared insertion site in both phenotypic lines.

To address this, we performed whole‐genome sequencing (WGS) using next‐generation sequencing (NGS) on individuals from both lines. Analysis of representative sequencing reads revealed divergence from the reference genome at position 41,006,965 on chromosome 10 in all reads from both lines, indicating a shared insertion site (Figure 6A,B). Similar results were obtained from the distal side of the breakpoint. To further confirm the shared insertion site identified by WGS, primers spanning the breakpoint were designed, and genomic PCR products were subjected to Sanger sequencing. The predicted breakpoint identified by WGS was successfully validated in both lines (Figure 6C–H).

In addition to confirming the insertion site, we reconfirmed the identity of the inserted transgene. In both lines, the transgene between *tol2* repeat elements included the rhodopsin promoter and the S334X mutant human rhodopsin expression cassette, which were found to be identical. The average sequencing depth at the insertion site was sufficient and comparable between the S334X‐benign (50.9×) and S334X‐original (48.3×) lines (Table 2), supporting the reliability of these findings and suggesting that both lines carry similar transgene copy numbers. Because the *tol2* transposon system typically integrates as a single copy without rearrangement and both lines share an identical insertion site and inheritance pattern, these findings collectively indicate that the transgene is present as a single‐copy insertion. Therefore, the observed phenotypic differences are not attributable to differences in transgene content or insertion site.

**TABLE 2:** Sequencing depth comparison between S334X‐benign line and original line.

Sample	Average depth (×)	Standard deviation (SD)	Region length (bp)	Covered bases (≥10×)	Coverage rate (%)
S334X‐original	48.31	8.06	401	401	100
S334X‐benign	50.95	11.92	401	393	98

Comparison of sequencing depth and coverage for the 401 bp insertion site in RHO:S334X‐original and RHO:S334X‐benign zebrafish lines. Average read depth, standard deviation (SD), number of bases covered at ≥10× depth, and overall coverage rate were calculated. Both lines exhibited high and comparable coverage, indicating that differences in phenotype are not attributable to variation in transgene coverage at the insertion site.

### 2.7 A *Cis*‐Regulatory Element Potentially Influencing Phenotype

Although the original and benign lines harbor the same S334X transgene at an identical genomic insertion site, they display divergent phenotypes. Because these phenotypes do not segregate across generations, the benign phenotype appears tightly linked to the transgene‐bearing allele. We therefore hypothesized that a *cis*‐regulatory element near the insertion site (Chr10: 41,006,965) attenuates transgene expression and contributes to the benign phenotype.

**FIGURE 7:**
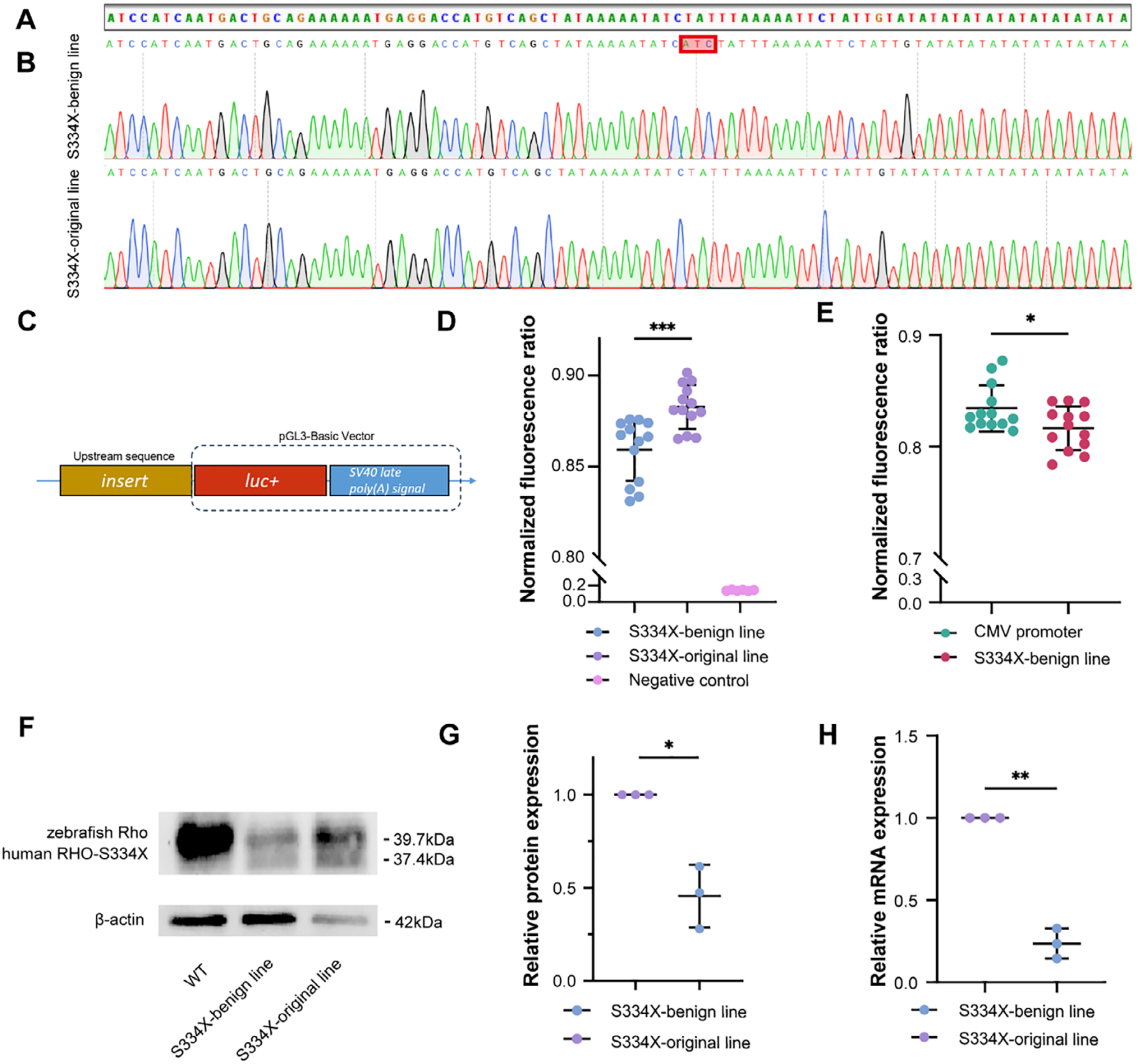
Functional and Expression‐Level Analysis of a Regulatory Sequence Variant Upstream of the *RHO*:S334X Transgene. Assessment of transcriptional activity of upstream sequences from benign and severe groups using luciferase reporter assays, Western blotting, and RT‐qPCR. (A) Reference genomic sequence corresponding to approximately 2.4 kb upstream region of the transgene insertion site (chr10:41 004 588), demonstrating the original sequence context (as observed in the S334X‐original line). (B) Cloning and sequencing of genomic DNA from the two lines confirms the 3‐bp insertion at chr10:41,004,588 in the S334X‐benign line (top panel). The corresponding region in the S334X‐original line lacks the insertion (bottom panel). (C) Schematic representation of luciferase reporter constructs containing ∼4.2 kb upstream regulatory regions cloned from S334X‐benign and S334X‐original zebrafish into the pGL3‐basic vector. (D) Luciferase activity measured at 48 h post‐transfection in HEK293T cells. The upstream region from the S334X‐benign line significantly represses luciferase expression compared to the S334X‐original construct, whereas the promoterless vector displayed only background signal. (***P < 0.001; unpaired t‐test; error bars represent SD, *n* = 13). (E) Luciferase activity measured at 48 h post‐transfection in HEK293T cells. The benign construct retained substantial promoter activity compared to the positive control. (*P < 0.05; unpaired t‐test; *n* = 12–13). (F) Representative Western blot analysis of rhodopsin expression in retinas from WT, S334X‐benign, and S334X‐original zebrafish. WT retinas express endogenous zebrafish rhodopsin (∼39.7 kDa), whereas both S334X‐benign and S334X‐original lines express endogenous zebrafish rhodopsin together with the human rhodopsin‐S334X protein (∼37.4 kDa). β‐actin (∼42 kDa) was used as a loading control. The expected molecular weights are indicated. (G) Quantification of relative human RHO‐S334X protein expression. Band intensities were first normalized to β‐actin and subsequently normalized to the S334X‐original line. Protein levels were significantly reduced in the S334X‐benign line compared with the S334X‐original line (*P < 0.05). Each data point represents one biological replicate, and error bars represent SD. Statistical significance was assessed using a paired two‐tailed Student's t‐test. (H) RT‐qPCR analysis of *RHO*: S334X transcript levels in the retina of S334X‐benign and S334X‐original zebrafish. Expression levels were normalized to endogenous *GAPDH* and quantified using the 2^−^ΔΔCt method (S334X‐original group set to 1.00). The S334X‐benign line exhibited significantly reduced expression levels (0.24 ± 0.09), corresponding to an approximately 4.2‐fold decrease (P = 0.0047, unpaired t‐test with Welch's correction). Error bars represent SD, *n* = 3 per group.

To test this, we isolated and compared ∼4.2 kb of upstream sequence specifically from the transgene‐bearing allele. To specifically analyze the transgene‐carrying allele, genomic regions flanking the insertion site were PCR‐amplified, cloned, and sequenced. The upstream region of approximately 4.2 kb spanning from the* tol2* left arm to the adjacent genomic sequence was fully sequenced in both lines. Sequence alignment confirmed that the entire 4.2 kb upstream region was identical between the benign and original lines, except for a distinct 3‐base pair (ATC) insertion located approximately 2.4 kb upstream of the breakpoint (position 41,004,588), which was present in the benign line but absent in the original line (Figure 7A, B).

These results indicate that the 3‐bp insertion represents the only sequence difference within the analyzed upstream regulatory region and may function as a *cis*‐regulatory variant contributing to the phenotypic divergence between the two lines.

### 2.8 Functional Validation of the *Cis*‐Regulatory Variant

To functionally assess whether the 3‐bp (ATC) insertion upstream of the transgene exerts a regulatory influence on transcription, we first performed in silico analyses of the genomic region flanking the insertion. Motif analysis did not predict the creation or disruption of any known transcription factor binding sites associated with the ATC insertion (Table S1), indicating that the regulatory effect of this variant is unlikely to be mediated through canonical sequence‐specific transcription factor binding.

Given recent evidence that local DNA shape features—such as minor groove width (MGW), helix twist (HelT), roll, and propeller twist (ProT)—can influence transcription independently of primary sequence motifs, we evaluated whether the ATC insertion induces localized alterations in DNA structure (Figure S3A–D). The predicted structural perturbations were confined to a narrow region immediately surrounding the insertion and were not observed in distal sequences, indicating that the ATC insertion is likely to modulate transcription by subtly altering local DNA geometry rather than inducing global structural changes.

Based on these in silico predictions, we next examined whether the 3‐bp insertion functionally affects expression of the pathogenic S334X allele using a luciferase reporter assay. Approximately 4.2 kb of upstream genomic sequence from both the benign and original lines were cloned into the pGL3 luciferase reporter vector (Figure 7C) and transfected into HEK293T cells to assess promoter activity.

The S334X‐benign construct exhibited significantly lower luciferase activity than the S334X‐original construct (*P* < 0.001), whereas the promoterless vector produced only background signal, confirming assay validity (Figure 7D). Notably, despite this attenuation, the benign construct retained substantial promoter activity comparable to that of the positive control CMV promoter (Figure 7E), indicating that the upstream region remains transcriptionally active but is specifically suppressed by the 3‐bp insertion.

Having established the *cis*‐regulatory impact of the ATC insertion in vitro, we next examined whether this regulatory effect is reflected in vivo. Western blot analysis revealed a marked reduction in S334X rhodopsin protein levels in the benign line compared with the original line (Figure 7F, G) (*P* < 0.05). Consistent with this reduction at the protein level, RT‐qPCR analysis of retinal cDNA demonstrated that RHO‐S334X transcript levels were significantly decreased in the benign line (0.24 ± 0.09) relative to the original line (set as 1.00), corresponding to an approximately 4.2‐fold reduction (*P* = 0.0047, Welch's t‐test) (Figure 7H).

Collectively, these in vitro and in vivo analyses demonstrate that the 3‐bp (ATC) insertion functions as a *cis*‐regulatory variant that attenuates transgene expression at both the transcriptional and protein levels, thereby contributing to the milder retinal degeneration phenotype observed in the benign line.

### 2.9 Segregation of a Severe Phenotype Derived from the Benign RP(S334X) Line

**FIGURE 8:**
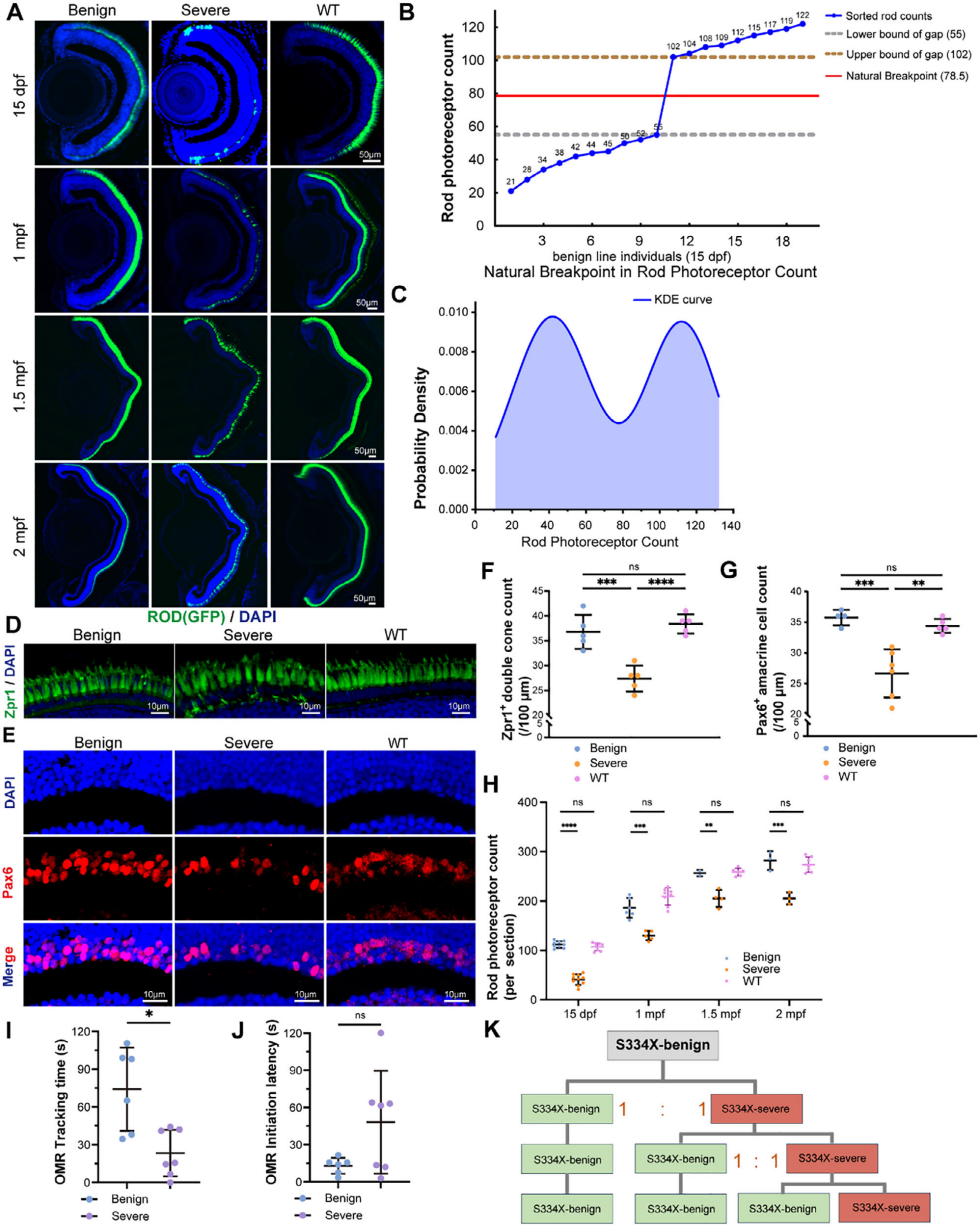
Phenotypic separation of zebrafish from the *RHO*:S334X‐benign line. Quantitative analysis of rod photoreceptor counts revealing bimodal distribution and Mendelian segregation in offspring from the benign line. (A) Retinal cryosections from S334X‐benign, S334X‐severe and WT zebrafish at 15 dpf, 1 mpf, 1.5 mpf, and 2 mpf: S334X‐benign showed rod photoreceptor survival comparable to WT, S334X‐severe demonstrating substantial photoreceptor loss. Rod photoreceptors are labeled with EGFP (green), and nuclei are counterstained with DAPI (blue). Scale bar = 50 µm. (B) Natural breakpoint analysis of 15 dpf rod photoreceptor counts revealed a clear discontinuity between 55 and 102, defining a breakpoint at 78.5 (red line). Sorted individual rod counts (Y‐axis) are plotted against individual zebrafish identifiers (X‐axis), where each X‐axis value represents a single S334X‐benign line individual ordered by increasing rod count. Dashed lines mark the lower and upper bounds of the observed phenotypic gap (gray: 55; brown: 102). (C) Kernel density estimation (KDE) of rod photoreceptor count distribution at 15 dpf. KDE uses a continuous smoothing kernel (Gaussian) and bandwidth selected via Silverman's rule of thumb to generate a smooth estimate of the underlying probability density function from discrete data points. The resulting KDE curve (blue shaded area) reveals a bimodal distribution, indicating that the trait is not continuous but dichotomous, with individuals segregating into either S334X‐benign or S334X‐severe phenotype. The local minima between the two peaks align closely with the natural breakpoint, providing independent, non‐parametric confirmation of phenotypic segregation. (D) Representative retinal cryosections from 15 dpf, S334X‐benign, S334X‐severe and WT stained with Zpr1 (green) and DAPI (blue). Scale bar: 10 µm. (E) Representative retinal cryosections from 15 dpf zebrafish, WT, S334X‐benign and S334X‐severe, stained for Pax6 (red) and DAPI (blue). Pax6^+^ cells are predominantly localized in the inner nuclear layer (INL), with occasional expression observed in the ganglion cell layer (GCL). INL, inner nuclear layer; IPL, inner plexiform layer; GCL, ganglion cell layer. Scale bar: 10 µm. (F) Quantification of double cones numbers shown as the number of Zpr1‐positive cells per 100 µm of retinal length (*n* = 5 per group). One‐way ANOVA followed by Tukey's multiple comparisons test (***P < 0.001; **P < 0.01; ns, not significant). (G) Quantification of amacrine cell numbers, shown as the number of Pax6‐positive cells in the INL per 100 µm of retinal length (*n* = 4–5 per group). One‐way ANOVA followed by Tukey's multiple comparisons test (****P < 0.0001; ***P < 0.001; ns, not significant). (H) Quantification of rod photoreceptor cell numbers in WT, S334X‐benign, and S334X‐sever at four developmental stages (15 dpf, 1 mpf, 1.5 mpf, and 2 mpf). Each data point represents an individual retina. Data are presented as mean ± SD (n ≥ 2 per group). Statistical comparisons were performed using one‐way ANOVA followed by Tukey's multiple comparisons test. ****P < 0.0001; ***P < 0.001; **P < 0.01; ns = not significant. (I, J) Quantification of tracking time (I) and initiation latency (J) during the optomotor response (OMR) assay in juvenile zebrafish (40 dpf; *n* = 13). OMR was first assessed in progeny derived from crosses between S334X‐severe fish and WT. Following behavioral testing, individual fish were labeled and subjected to post hoc retinal cryosectioning. Based on rod photoreceptor counts, animals were subsequently classified into S334X‐benign (*n* = 6) and S334X‐severe (*n* = 7) groups, and tracking time and initiation latency were analyzed accordingly. Statistical significance was assessed using a two‐tailed Mann–Whitney U test (*P < 0.05; ns, not significant). Error bars represent SD. (K) Schematic representation of phenotypic outcomes observed in offspring from different Tg(hs.*RHO*:S334X)‐benign line zebrafish. In some families, all progeny retained the benign phenotype (S334X‐benign). In others, the progeny segregated into two distinct phenotypic categories: a benign group and a severe phenotype group, and the ratio of the number of individuals of the two phenotypes is approximately 1 to 1.

The RP(S334X) transgene is fully linked to RP onset, and a unique 3‐bp insertion near its integration site is present exclusively in the benign line, which has been demonstrated to contribute to its attenuated phenotype. However, in the third generation derived from the benign G0 founder, a subset of offspring unexpectedly exhibited a severe retinal degeneration phenotype, despite originating from a lineage previously classified as benign (Figure 8A). Notably, both benign and severe phenotypes were observed among siblings from the same cross, indicating that the benign phenotype was no longer uniformly expressed. This unexpected phenotypic segregation prompted us to investigate whether the variation represented a continuous spectrum or a dichotomous trait. To address this, we performed quantitative analyses of rod photoreceptor counts.

Natural breakpoint analysis of rod photoreceptor counts at 15 dpf revealed a clear gap (55–102 cells/section), with a breakpoint at 78.5 cells/section, separating individuals into two clearly defined clusters (Figure 8B). Kernel density estimation (KDE) and k‐means clustering (k = 2) confirmed a bimodal distribution, corresponding to benign and severe phenotypes (Figure 8C; Figure S4), indicating that the trait is not continuous but dichotomous, with individuals clearly segregating into either a ‘benign’ or ‘severe’ phenotype. Having established a clear dichotomous segregation based on rod photoreceptor counts at 15 dpf, we further characterized the retinal phenotype of individuals classified as severe at this time point. In addition to markedly reduced rod numbers, double cone counts were significantly lower in the severe group compared with both the mild group and WT controls, whereas no significant difference was observed between the benign and WT groups. Amacrine cell numbers similarly remained comparable between benign and WT retinas, but were reduced specifically in severe individuals (Figure 8D–G). Together, these observations further support the robustness of the rod‐based phenotypic segregation observed at 15 dpf.

To determine the developmental onset of this segregation, similar analyses were performed at 1, 1.5, and 2 mpf, yielding consistent breakpoints (150.5, 239, and 239.5 cells/section, respectively; Figure S5A–C). At all timepoints, the severe group exhibited significantly reduced rod counts compared to both benign and WT groups. Rod counts in the benign group were statistically indistinguishable from WT controls, reinforcing the physiological normality of the benign phenotype (Figure 8H).

Consistent with this dichotomous structural segregation, optomotor response (OMR) assays at 40 dpf revealed significantly longer tracking time in the benign group than in the severe group, whereas initiation latency did not differ between groups (Figure 8I, J). Rod photoreceptor counts were positively correlated with OMR tracking time at 40 dpf (r = 0.75), and remained weakly correlated at 80 dpf (r = 0.31) (Figure S6). These behavioral data provide functional support for the rod‐based phenotypic segregation. Sequence analysis confirmed that the RP(S334X) transgene was identical in both phenotypic groups (data not shown), ruling out transgene variation as the cause of the observed segregation.

Together, these structural, cellular, and behavioral findings demonstrate that the benign lineage segregates into two discrete phenotypic classes—benign and severe—independently of the S334X transgene itself. This strongly suggests the presence of an additional genetic factor modulating disease severity, setting the stage for subsequent segregation and inheritance analyses.

### 2.10 Segregation Analysis Reveals a Dominant *Trans*‐Acting Modifier

The emergence of a dichotomous phenotype within the benign RP(S334X) lineage suggested the involvement of a genetic modifier. Based on Mendelian principles, if a single dominant modifier gene segregates independently of the RP(S334X) transgene, and the transgenic parent is heterozygous for this modifier, we would expect a 1:1 ratio of benign to severe phenotypes among the offspring.

To test this, transgenic fish were crossed with WT individuals from a different lineage. Because rod photoreceptor quantification requires post‐mortem analysis, the phenotype of the transgenic parent could not be determined prior to mating. As a result, parental phenotype was retrospectively inferred from the phenotypes of the offspring. These crosses resulted in two distinct progeny groups: one in which offspring segregated into both benign and severe phenotypes, and another in which all offspring exhibited only the benign phenotype. This phenotypic dichotomy supports the hypothesis of a single dominant *trans*‐acting modifier (Figure 8K). For crosses that exhibited segregation, we specifically analyzed the ratio of benign to severe individuals among the offspring. Fisher's exact test showed no deviation from the expected 1:1 ratio at any timepoint, and pooled data confirmed this by chi‐square test (χ^2^ = 0.049, *P* = 0.8252; Table 3). Sequence analysis confirmed that both phenotypic groups carried an identical RP(S334X) transgene (data not shown), ruling out variation within the transgene itself as the cause of the observed 1:1 segregation.

**TABLE 3:** Ratio of benign to severe individuals among the offspring of benign line.

Time point	Benign	Severe	Statistical test	P‐value
15dpf	9	10	Fisher's exact	1.0000
1mpf	6	5	Fisher's exact	1.0000
1.5mpf	2	4	Fisher's exact	0.6379
2mpf	3	3	Fisher's exact	1.0000
**Total**	20	22	Chi‐square test	0.7371

Distribution of the two phenotypes (benign vs. severe) within the S334X‐benign line at each developmental stage. Phenotypic segregation was assessed at four developmental stages (15 dpf, 1 mpf, 1.5 mpf, and 2 mpf). Due to the small sample sizes at individual time points, Fisher's exact test was applied to evaluate whether the observed distribution deviated from the expected Mendelian 1:1 ratio. For the pooled data across all time points, a chi‐square test was used. No significant deviation from the expected 1:1 segregation ratio was observed at any time point or in the cumulative analysis, supporting the hypothesis that a single dominant‐acting modifier segregates independently of the RP(S334X) transgene.

To determine whether the severe phenotype arose from loss of the 3‐bp (ATC) insertion associated with the benign line, we examined the insertion status across different phenotypic groups. The original line consistently lacked the ATC insertion, whereas both benign and severe individuals retained the ATC insertion (Figure S7).

Collectively, these findings indicate that the observed phenotypic segregation is driven by a dominant genetic factor that operates independently of the S334X transgene. The RP phenotype induced by the S334X transgene appears to be mitigated by a *cis‐*regulatory element—specifically, a 3‐bp insertion located just upstream of the transgene integration site. However, this protective effect can be overridden or exacerbated by a *trans*‐acting modifier located elsewhere in the genome.

## 3 Discussion

In inherited disorders with broad phenotypic diversity, even members of the same family who carry an identical disease‐causing allele can exhibit markedly different disease severities. RP is a representative example. These long‐standing clinical observations have implied roles for factors beyond environment, most plausibly genetic contributors acting outside the primary allele, yet direct experimental evidence has been scarce [6, 27, 28]. In this study, within a single vertebrate model in which the same pathogenic allele (*RHO* S334X) resides at the same genomic insertion site, we show that two independent mechanisms operate in vivo to modulate phenotypic severity. Specifically, (i) a 3‐bp (ATC) insertion located ∼2.4 kb upstream of the integration site acts as a protective *cis*‐regulatory variant that attenuates transgene expression and yields a benign phenotype, and (ii) a dominant *trans*‐acting modifier, segregating at an approximately 1:1 Mendelian ratio in specific families, converts this benign state to a severe phenotype. Together, these findings provide direct experimental support for a dual genetic regulatory architecture of RP phenotypic heterogeneity—a concept long hypothesized but never before empirically demonstrated within a single, controlled vertebrate model [6, 7, 27].

The benign phenotype harbors a unique 3‐bp (ATC) insertion in the upstream genomic region of the transgene (Figure 7A,B), absent from the severe phenotype while the transgene sequence and genomic insertion site are otherwise identical between lines (Figures 5 and 6). Functionally, the benign upstream fragment reduces reporter activity in luciferase assays and lowers *RHO* S334X transcript and protein levels in vivo (Figure 7C–G), thereby supporting a parsimonious mechanism for phenotypic amelioration through expression attenuation. Motif scanning did not support gain/loss of canonical transcription factor binding sites; instead, in silico DNA‐shape analysis (minor groove width, helix twist, roll, propeller twist) indicated localized alterations confined to the vicinity of the insertion (Figure S3A–D). These observations are consistent with the notion that DNA structural features, independent of primary sequence motifs, contribute to transcriptional regulation by modulating protein–DNA recognition and chromatin architecture [29, 30, 31]. While the reporter assays were performed in a heterologous cell context, the direction of effect replicated in vivo by RT‐qPCR and Western blot strengthens the inference that the upstream insertion attenuates transgene expression. Within the specific pathophysiologic context of class I rhodopsin mutations that abolish the C‐terminal VxPx trafficking signal (e.g., S334X, Q344X), even modest reductions in the dosage of mislocalized rhodopsin can meaningfully diminish downstream cellular stress and degeneration [20, 28, 32, 33, 34, 35]. Thus, our data support a model in which a “microstructural” change in DNA encodes a “macro‐phenotypic” protection by dialing down the expression of a toxic allele.

In the third generation derived from the benign founder, rod counts segregated into two discrete clusters with a natural breakpoint, yielding a bimodal distribution confirmed by kernel density estimation and *k*‐means clustering (Figure 8B–C; Figure S4). The severe subset retained the ATC insertion (Figure S7), ruling out loss of the *cis* variant as the cause. Across independent families, the severe phenotype segregated at an approximately 1:1 ratio from specific parental crosses (Table 3), a pattern consistent with a single dominant *trans*‐acting modifier operating independently of the S334X transgene. Such a genetic architecture is biologically plausible in zebrafish populations that harbor substantial standing genetic variation—including abundant copy‐number variation and substructure—and are inherently outbred, even in commonly used laboratory lines [36, 37]. Routine outcrossing, as used here, preserves this background diversity and can unmask large‐effect modifiers in a “natural experiment” setting that would be difficult to engineer in inbred mammalian models. The existence of a dominant modifier that can override a protective *cis* variant highlights a layered regulatory logic: the ultimate disease trajectory is determined by the composition of *cis* features at the locus and the state of *trans* regulators across the genome.

Clinically and genetically, RP is renowned for both locus heterogeneity and intra‐allelic variability, the latter being repeatedly documented since the earliest discoveries of *RHO* mutations in autosomal dominant RP [6, 7, 27]. Yet, direct experimental dissection of both *cis* and *trans* influences under an identical pathogenic allele and within an identical genomic context has been lacking. Our study fills this gap by integrating (i) molecular identification of the transgene and its insertion site (Figures 5 and 6), (ii) functional validation of upstream regulatory activity and in vivo expression levels (Figure 7), and (iii) classical segregation analysis of the severe phenotype (Figure 8). Conceptually, these elements converge on a dual‐regulatory framework: a protective *cis* variant dampens toxic gene dosage, while a dominant *trans* factor can restore or exacerbate pathology despite *cis* protection. This framework provides a concrete mechanistic basis for interpreting the intrafamilial variability frequently encountered in RP clinics, where the same mutation may present with divergent rates of progression and functional impairment [7, 11].

Although RP is classically rod‐initiated, accumulating clinical evidence indicates that cone dysfunction can emerge early even when outer‐segment structure is partly preserved, as revealed by optoretinography and adaptive‐optics imaging [33, 34]. In this light, the preserved cone and inner‐retinal populations together with maintained OMR performance in the benign line provide a functional parallel to “hypomorphic” clinical presentations reported for atypical *RPGR* splice variants [35, 38]. These observations align our zebrafish findings with human variability while underscoring that clinical generalizability remains to be determined and should be interpreted cautiously.

The dominant *trans* modifier identified here does not trigger RP in the absence of the pathogenic background, indicating that *cis* and *trans* contributions are genetically independent yet functionally interdependent in the same context. Two practical questions remain. First, does the *trans* modifier primarily aggravate disease by changing the effective dosage (expression) of the pathogenic allele—potentially intersecting with the *cis*‐encoded attenuation—or does it act through a separate pathway (e.g., proteostasis burden, phototransduction load, chromatin state) that does not require expression changes? Second, is the observed aggravation allele‐specific (e.g., uniquely sensitive to the 3‐bp ATC context and its DNA‐shape consequences) or generalizable across RP genotypes?This split materially affects the value of the model. If generalizable, the *trans* factor would represent a key molecule in retinal protection/injury and a broad therapeutic target, and it may become an important stratification factor in both basic and translational RP studies. If allele‐specific, it still provides a clear mechanistic model for a TF‐independent link between local DNA structure and gene expression, a domain that has been difficult to dissect with motif‐based reasoning alone [29, 30, 31]. In all cases, molecular identification of the *trans* modifier is a high priority. Alongside linkage analysis leading to positional cloning, we have initiated dosage‐sensitive measurements to determine whether the *trans* effect converges on expression control: allele‐specific expression (ASE) in photoreceptors, tiered RT‐qPCR/Western blot quantification, and ATAC‐seq/ChIP‐seq to profile chromatin accessibility and TF occupancy. These data will rigorously adjudicate between dosage‐based vs. orthogonal mechanisms and between allele‐specific vs. generalizable effects in controlled systems.

An identical disease allele is not destiny—in any disorder with a genetic component. Phenotypic trajectories are jointly shaped by a *cis*‐regulatory layer—local DNA shape and chromatin context controlling dosage—and a *trans*‐regulatory layer—modifier states distributed across the genome. Demonstrating both layers under the same allele and the same genomic context moves the field from conjecture to experimentally grounded logic with implications beyond retinopathies [11, 30]. Practically, this dual‐regulatory view suggests two complementary levers for precision medicine: attenuating toxic dosage at the locus and modulating background modifier states. While population and human‐genetics approaches remain indispensable, they often cannot resolve allele‐level *cis–trans* interactions and context‐dependent regulation, underscoring the value of controlled models that fix the primary allele and its position to deconvolute modifier mechanisms [36, 37].

## 4 Methods

### 4.1 Sex as a Biological Variable

Our study examined male and female zebrafish, and similar findings are reported for both sexes.

### 4.2 Animals and Study Approval

All zebrafish experiments were conducted in accordance with the ARVO Statement for the Use of Animals in Ophthalmic and Vision Research. Zebrafish (Danio rerio, AB strain) were housed and bred under standard conditions, and embryonic staging was performed as previously described [39, 40]. Genetic modification of zebrafish was approved by the Genetic Modification Safety Committee of Osaka University (Approval No. 05306).

### 4.3 Generation and Identification of Transgenic Zebrafish

Transgenic zebrafish were generated using the *tol2* transposon system [41]. The S334X mutant human *rhodopsin* gene, driven by the human *rhodopsin* (*RHO*) promoter, was subcloned into expression vectors. To facilitate identification of transgenic individuals, a GFP reporter gene under the control of the olfactory marker protein (OMP) promoter was inserted in parallel between the *tol*2 arms. This olfactory expression cassette enabled us to identify transgene‐positive fish based on nasal fluorescence. The expression construct was microinjected into one‐cell‐stage zebrafish embryos along with *tol*2 transposase mRNA. Stable transgenic lines were selected based on nasal GFP fluorescence, followed by genotyping using PCR and direct sequencing of the transgene cassette (Figure S8A,B).

### 4.4 Outcrossing Strategy and Experimental Context

The S334X transgenic zebrafish line used in this study has been maintained in our laboratory for approximately 12 years. To minimize inbreeding and reduce the accumulation of background mutations, the line was maintained by routine outcrossing to AB wild‐type zebrafish at each generation. The AB strain is a widely used reference wild‐type line in zebrafish research, characterized by a well‐defined and relatively homogeneous genetic background. Continuous outcrossing to AB wild‐type fish allows stable transmission of the S334X transgene while repeatedly resetting the genetic background toward a standardized reference genome. This strategy effectively limits genetic drift and suppresses the fixation of spontaneous or recessive deleterious variants that may arise during long‐term colony maintenance.

Importantly, routine outcrossing preserves genetic diversity at non‐transgenic loci while maintaining consistent expression of the pathogenic S334X allele, thereby enabling reliable assessment of genotype–phenotype relationships across generations. As a result, phenotypic differences observed in this study are unlikely to reflect nonspecific effects of prolonged inbreeding or colony instability, but instead arise from defined genetic factors segregating within the outcrossed population.

All zebrafish, including both benign and severe S334X‐derived groups, were maintained under identical husbandry conditions throughout the study. Fish were housed in the same recirculating aquaculture system under a standard light–dark cycle, temperature, feeding regimen, and population density, following established zebrafish husbandry guidelines. No selective breeding or differential maintenance was applied based on phenotypic appearance.

Notably, the benign phenotype emerged abruptly from a single founder despite long‐term uniform rearing conditions, arguing against preferential preservation of either phenotype by husbandry or environmental factors.

### 4.5 Histological and Immunofluorescence Analysis of Retinal Neurons in Transgenic Zebrafish

To facilitate visualization and quantification of rod photoreceptor cells, S334X transgenic zebrafish were crossed with a previously characterized zebrafish line expressing enhanced GFP (EGFP) under the control of the zebrafish *rhodopsin* promoter *(rh1*) (Figure S8B). This *rh1*:EGFP zebrafish line was gifted by Dr. Kawamura [42]. Offspring exhibiting both nasal and ocular GFP fluorescence were classified as the RP group, while individuals showing only ocular fluorescence were used as WT controls.

For rod cell quantification, embryos were fixed in 4% paraformaldehyde (PFA, w/v, pH 7.4) overnight at 4°C, and then dehydrated in 30% sucrose. Tissue embedding and cryosectioning were performed as previously described. Retinal sections were cut at 13 µm (5, 7, 15 dpf), 25 µm (1, 1.5 mpf), and 30 µm (2, 3 mpf) along the lens–optic nerve axis, using one section per eye. Embryonic and adult retinal phenotypes were examined using a Zeiss Axioscope microscope or a Bio‐Rad Radiance 2100 confocal microscope.

For immunofluorescence analysis of cone photoreceptors, amacrine cells, and inner retinal organization, cryosections were washed three times for 10 min each in phosphate‐buffered saline (PBS) and then incubated in blocking solution containing PBS supplemented with 10% bovine serum albumin (BSA) and 0.3% Triton X‐100 for 1 h at room temperature. Sections were subsequently incubated overnight at 4°C with primary antibodies against Zpr1, Pax6, or Calbindin. After washing three times in PBS, appropriate species‐specific secondary antibodies conjugated to Alexa Fluor 568 or Alexa Fluor 647 were applied for 1 h at room temperature. Following three additional PBS washes, nuclei were counterstained with 4′,6‐diamidino‐2‐phenylindole (DAPI) for 10 min at room temperature. Stained sections were mounted and imaged using confocal microscopy under identical acquisition settings for comparative analysis.

### 4.6 Quantification of Retinal Neurons in Cryosections

Images were acquired using an OLYMPUS FV1200 laser scanning confocal microscope. Image processing was performed using FV10‐ASW 4.2 Viewer or Adobe Photoshop software (Adobe Inc.) for visualization and cell counting. Rod photoreceptors were identified as GFP‐positive cells and manually counted in the central retinal region by two independent, blinded observers. Statistical comparisons between transgenic lines were performed using unpaired t‐tests or one‐way ANOVA followed by Tukey's multiple comparisons test.

For immunofluorescence‐stained sections used in cone photoreceptor, amacrine cell, and inner retinal analyses, confocal images were acquired as Z‐stacks with a step size of 5 µm under identical imaging settings across experimental groups. Maximum intensity projections were generated for both quantitative and qualitative analyses.

For cone photoreceptor quantification, three non‐overlapping regions of interest (each 100 µm in length) were selected from the central retina of each section, and double cone numbers were counted within each region and averaged to obtain a single value per eye. For amacrine cell quantification, positive cells were counted along the inner plexiform layer (IPL) and expressed as the number of cells per 100 µm length of IPL. All quantifications were performed by two independent observers in a blinded manner.

### 4.7 Genomic DNA Extraction and PCR Analysis

Genomic DNA (gDNA) was extracted from zebrafish tissue using the QIAGEN DNeasy Blood & Tissue Kit (QIAGEN, Germany) according to the manufacturer's instructions. The concentration and purity of the extracted gDNA were assessed using a NanoDrop 2000 spectrophotometer (Thermo Fisher Scientific).

PCR was performed using specific primers targeting the *RHO* transgene and its flanking genomic regions (Table S2). Reaction conditions were optimized to ensure specific amplification. PCR products were separated by 1.5% agarose gel electrophoresis and visualized under UV light.

### 4.8 Whole‐Genome Sequencing and Mapping of Transgene Insertion Sites

Whole‐genome sequencing (WGS) was performed using the Illumina platform (Rhelixa, Japan). Raw sequencing data in FASTQ format were quality‐checked and aligned to the GRCz11 zebrafish reference genome using BWA. Format conversion and processing (e.g., FASTQ to BAM) were performed using standard bioinformatics tools.

To identify transgene insertion sites, sequencing reads were manually inspected using UltraEdit to screen for junction reads containing both genomic and vector‐derived sequences. These reads were visualized using IGV version 2.16. Junction read analysis revealed that the S334X transgene was inserted at Chromosome 10: 41 006 965 in both transgenic zebrafish lines. Similarly, the *rh1*:EGFP transgene was mapped to Chromosome 3: 46 902 754.

### 4.9 Confirmation of the *Cis*‐Regulatory Element in the S334X‐Benign Line

To determine whether the 3‐base pair (ATC) insertion identified upstream of the transgene by whole‐genome sequencing is located on the same chromosome as the integrated transgene, we performed PCR walking targeting the entire upstream region. Primers were designed to amplify genomic fragments containing informative single‐nucleotide variants (SNVs) that distinguish paternal and maternal alleles. Overlapping PCR products were generated to span the entire target region, enabling the construction of a contig.

PCR amplification was carried out using a high‐fidelity polymerase (KOD One, TOYOBO), and the resulting products were cloned into the ZERO‐BLUNT vector. All clones were subjected to Sanger sequencing. Based on the obtained sequence data, we reconstructed the haplotype of the chromosome harboring the transgene by referencing whole‐genome sequencing results.

This analysis confirmed that the S334X‐benign line harbors a three‐nucleotide insertion (ATC) upstream of the transgene integration site, whereas the S334X‐original line retains the WT sequence.

### 4.10 RT‐PCR and Quantitative Real‐Time PCR (RT‐qPCR)

Total RNA was extracted from zebrafish retinas using ISOGEN II reagent (NIPPON GENE, Japan), and cDNA was synthesized using a reverse transcription kit (Takara Bio, Japan). RT‐qPCR was performed using SYBR Green Master Mix (Thermo Fisher Scientific, USA) on a QuantStudio 5 Real‐Time PCR System (Applied Biosystems, USA).Relative gene expression levels were normalized to GAPDH and calculated using the 2^–ΔΔCt method. The primer sequences used are listed in Table S3.

### 4.11 Western Blot Analysis

Retinas from 5 mpf zebrafish, including WT, S334X‐original, and S334X‐benign lines, were randomly selected for protein analysis. For each line, retinas from three individual fish were pooled as independent biological replicates. Total protein was extracted using standard lysis procedures, and protein concentrations were determined prior to electrophoresis.

Equal amounts of protein (20 µg per lane) were separated on Mini‐PROTEAN TGX precast gels (Bio‐Rad) and subsequently transferred onto polyvinylidene difluoride (PVDF) membranes using the Trans‐Blot Turbo Transfer System (Bio‐Rad). A prestained molecular weight marker (4–20% TG SDS buffer marker; Proteinark) was used to monitor protein separation and transfer efficiency.

Membranes were blocked in blocking buffer for 30 min at room temperature and then incubated overnight at 4 °C with primary antibodies against rhodopsin (anti‐rhodopsin, clone 1D4; Abcam) or β‐actin (loading control). After washing, membranes were incubated with appropriate species‐specific horseradish peroxidase–conjugated secondary antibodies (Cell Signaling Technology) at room temperature. Protein signals were detected using enhanced chemiluminescence reagents and visualized with an automated gel imaging system. Western blot band intensities were quantified using ImageJ software (National Institutes of Health). The integrated density of each target band was measured following background subtraction and normalized to the corresponding β‐actin band from the same lane. Only nonsaturated signals within the linear range of detection were used for quantification. Data were obtained from independent biological replicates.

### 4.12 Motif Analysis

To evaluate whether the ATC insertion is associated with the gain or loss of canonical transcription factor binding sites, motif analysis was performed on genomic sequences flanking the insertion site. Sequences encompassing the ATC insertion in the S334X‐benign groups and the corresponding region from the S334X‐original groups were analyzed using the JASPAR database [43] and position weight matrix (PWM)–based scanning.

Motif scanning was conducted using default background nucleotide frequencies, and predicted transcription factor binding sites were identified based on relative profile score thresholds recommended by the JASPAR database. Only motif matches exceeding a relative score threshold of 95% were considered for further analysis. Comparative motif profiles between the benign and severe alleles were examined to assess potential motif gain or loss associated with the ATC insertion.

Within the limits of motif‐based prediction, no creation or disruption of known canonical transcription factor binding motifs was detected in the vicinity of the ATC insertion.

### 4.13 DNA Shape Prediction

DNA structural features were predicted using the DNAshape framework, which estimates sequence‐dependent DNA shape parameters based on a pentamer‐based model [29]. Genomic sequences encompassing the ATC insertion site were extracted from both the benign and original alleles and subjected to DNA shape analysis. Four DNA shape features were calculated, including minor groove width (MGW), helix twist (HelT), roll, and propeller twist (ProT).

Predicted DNA shape values were visualized as continuous profiles across the analyzed sequence window. Positions for which DNA shape parameters could not be computed due to algorithmic constraints at sequence boundaries were treated as missing values. DNA shape profiles were compared between the benign and original alleles without artificial padding or sequence modification.

### 4.14 Cell Culture

Human embryonic kidney (HEK) 293T (CVCL_0063) cells were obtained from ATCC (American Type Culture Collection, Manassas, VA, USA) in 2006 and have been maintained in our laboratory since that time. The cells were routinely tested negative for mycoplasma contamination by PCR. Cells were cultured in Dulbecco's Modified Eagle Medium (DMEM; Gibco) supplemented with 10% fetal bovine serum (FBS; Gibco) and 1% penicillin/streptomycin. Cells were cultured in 10‐cm dishes and detached using 0.25% trypsin‐EDTA (v/v) in PBS when confluency reached approximately 80%.

### 4.15 Cell Transfection and Luciferase Assay

Luciferase assays were performed using the Dual‐Glo Luciferase Assay System (Promega, E2920) according to the manufacturer's instructions. A Renilla luciferase plasmid was co‐transfected to normalize transfection efficiency. Upstream sequences from the benign and severe groups were excised using restriction enzymes and ligated into the pGL3‐Basic Vector (Promega) with DNA ligase (Ligation Solution I). Positive clones were screened, and plasmids were extracted for luciferase assays. The pGL3‐Control Vector served as a positive control and the promoterless vector (pGL3‐Basic) as a negative control.

HEK293T cells were seeded in black‐walled 96‐well plates and transfected using Lipofectamine 3000 (Invitrogen, USA) in Opti‐MEM medium when confluency reached ∼80% (day 2–3 post‐seeding). After 6 h, the medium was replaced with DMEM containing 10% FBS and 1% penicillin/streptomycin. Luciferase activity was measured at 36 and 48 h post‐transfection using the Dual‐Glo Reporter System.

### 4.16 Optomotor Response (OMR) Assay

We conducted the OMR test using a system similar to that reported previously [26]. A motor and an electronic control system were used to rotate black and white stripes (1.1 cm in width) around an inner cylindrical acrylic tank (diameter = 14.0 cm). The rotation speed was controlled between 15–20 rpm, and the rotation direction could be switched. According to the growth stage of the zebrafish, the optimal tracking speed was set at 15 rpm for 40 dpf fish and 20 rpm for 80 dpf fish. Data were recorded from beneath the cylinder using a video camera. Prior to behavioral testing, fish were dark‐adapted for at least 30 min in a light‐tight container to standardize retinal sensitivity and minimize the effects of prior light exposure. After dark adaptation, the fish was introduced into the cylindrical tank. The assay was conducted under controlled ambient illumination. It was allowed to remain stationary for 1 min for acclimation. The stripe‐background was then rotated clockwise for 2 min while recording the fish's movement. After an additional 1‐min resting period, the wheel was rotated counterclockwise for 2 min to measure the fish's behavior. Tracking behavior was defined as sustained swimming in the same direction as the rotating stripes with a swimming velocity equal to or greater than 50% of the stripe rotation speed. Two behavioral parameters were quantified: tracking time, defined as the cumulative duration during which the fish swam in the same direction as the rotating stimulus and response latency, defined as the time from stimulus onset to the initiation of the first sustained tracking movement. Tracking time and latency were measured separately for clockwise and counterclockwise rotations. Mean values across both directions were calculated for descriptive purposes. The acquired data were quantified using a previously reported analysis program [44]. Statistical analyses were performed using GraphPad Prism. Due to non‐normal distribution and heterogeneity of variance, non‐parametric tests were applied. Comparisons among multiple groups were conducted using the Kruskal–Wallis test followed by Dunn's multiple comparisons test when appropriate. Correlations between behavioral parameters and rod photoreceptor counts were evaluated using Pearson or Spearman correlation analysis. *P* < 0.05 was considered statistically significant.

### 4.17 Natural Breakpoint Analysis, KDE, and K‐Means Clustering

Rod photoreceptor counts were sorted in ascending order. The natural breakpoint was defined as the midpoint of the largest numerical gap between adjacent values in the sorted distribution. Breakpoint values were determined using Python (version 3.10), and data were visualized using GraphPad Prism.

Kernel density estimation (KDE) was used to evaluate the distribution of rod photoreceptor counts and assess bimodal patterns. KDE was computed in Python using a Gaussian kernel with bandwidth selected by Silverman's rule of thumb. Smoothed density curves were plotted using OriginPro based on KDE results exported from Python.

K‐means clustering (k = 2) was performed to classify individuals into two groups based on rod photoreceptor counts. Clustering was implemented in Python using the scikit‐learn library, and cluster assignments were visualized using GraphPad Prism.

### 4.18 Statistical Analysis

All experiments were independently repeated at least three times using biologically independent samples. Data were presented as mean ± standard deviation (SD). Statistical analyses were performed using GraphPad Prism (version 9.0).

For comparisons between two independent groups, unpaired two‐tailed Student's t‐tests were used when data satisfied assumptions of normality and homogeneity of variance. Paired two‐tailed Student's t‐tests were applied when comparisons were made between matched or paired samples. When data did not meet parametric assumptions, the two‐tailed Mann–Whitney U test was used.

For comparisons involving more than two groups, one‐way analysis of variance (ANOVA) followed by Tukey's multiple comparisons test was applied for normally distributed data. For non‐normally distributed data, the Kruskal–Wallis test followed by Dunn's multiple comparisons test was used.

Statistical significance was defined as *P* < 0.05 (*P < 0.05; **P < 0.01; ***P < 0.001; ****P < 0.0001; ns, not significant). Sample sizes (n), representing biological replicates, are indicated in the corresponding figure legends. No data were excluded from the analyses. Investigators were blinded to group allocation during image acquisition and quantitative analysis.

## Funding

This work was supported by JSPS KAKENHI Grants (JP25K02794, JP24K22167, and JP23K21480) and by AMED Grant JP24gm1510010h.

## Conflicts of Interest

The authors declare no conflict of interest.

The dataset and analytic code used to generate the KDE curve in Figure X are available in the following public GitHub repository: https://github.com/VIitor0831/RP_KDE_analysis.git (commit ID: b2988af).

The repository includes the original rod photoreceptor cell counts used for the analysis and the Python script for performing the kernel density estimation (KDE).

Additional data are available from the corresponding author upon reasonable request. Whole‐genome sequencing data have been deposited in the DNA Data Bank of Japan (DDBJ) Sequence Read Archive under DDBJ BioProject Accession number **PRJDB35936**, DRA accession number **DRA021927** and Run accession numbers **DRR720097** and **DRR720098**. Public release of the deposited data has been requested through DDBJ.

The released project(s) is available in the DDBJ Search https://ddbj.nig.ac.jp/search.

The released project(s) will also be available at the NCBI BioProject http://www.ncbi.nlm.nih.gov/bioproject/.

## Supporting information




**Supporting File 1**: advs75176‐sup‐0001‐SuppMat.docx.

**Supporting File 2**: advs75176‐sup‐0002‐Video S1.mp4.

**Supporting File 3**: advs75176‐sup‐0003‐Video S2.mp4.

**Supporting File 4**: advs75176‐sup‐0004‐Video S3.mp4.

**Supporting File 5**: advs75176‐sup‐0005‐Video S4.mp4.

**Supporting File 6**: advs75176‐sup‐0006‐Video S5.mp4.
[Correction added on 22 April 2026 after first online publication: the supporting information video files have been arranged in order in this version.]

## Data Availability

All data supporting the findings of this study are available within the article and its Supporting Information files.
