# Supplementary material for: Cis‐ and Trans‐Regulatory Factors Independently Shape Phenotypic Heterogeneity of Retinitis Pigmentosa
Source: Adv Sci (Weinh). 2026 Apr 10;13(36):e20828. doi: 10.1002/advs.202520828 (PMC13317566; doi:10.1002/advs.202520828)
Supplement: Supplementary file 1 — Supporting File 1: advs75176‐sup‐0001‐SuppMat.docx. [file ADVS-13-e20828-s002.docx]

‘**Supplementary Figure 1**


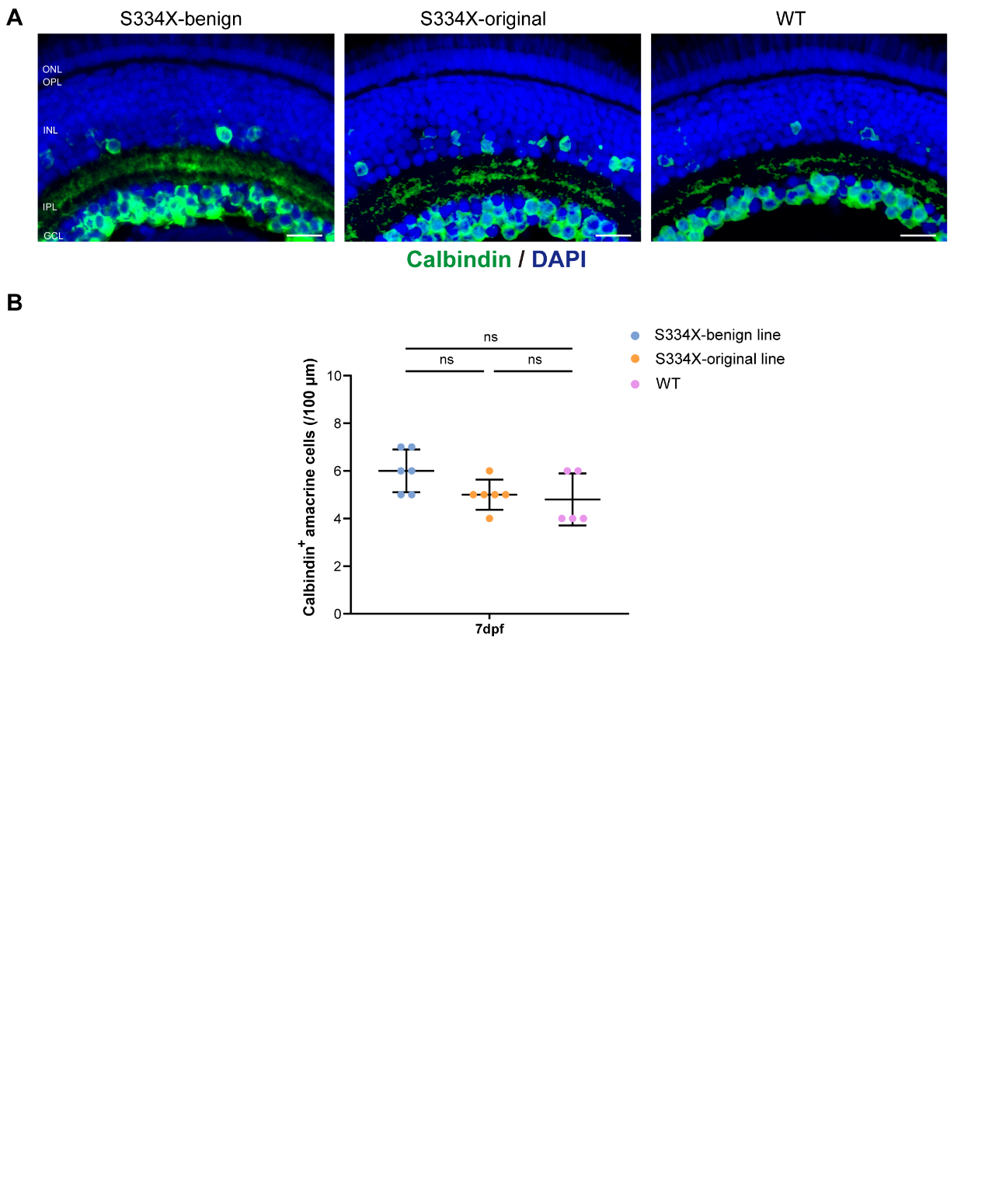


**(A)**Representative retinal cryosections(7dpf) from S334X-benign, S334X-original line and WT zebrafish stained for Calbindin (green), with nuclei counterstained by DAPI (blue). Calbindin-positive amacrine cells are localized to the inner nuclear layer (INL). The overall laminar organization of the IPL is preserved and comparable among WT, original, and benign retinas, with no apparent structural differences observed. ONL, outer nuclear layer; OPL, outer plexiform layer; INL, inner nuclear layer; IPL, inner plexiform layer; GCL, ganglion cell layer. Scale bars, 10 μm. **(B)** Quantification of Calbindin⁺ cells in the INL, shown as the number of Calbindin-positive cells per 100 μm of retinal length (n = 5-6 per group). One-way ANOVA followed by Tukey’s multiple comparisons test (ns, not significant)

**Supplementary Figure 2**


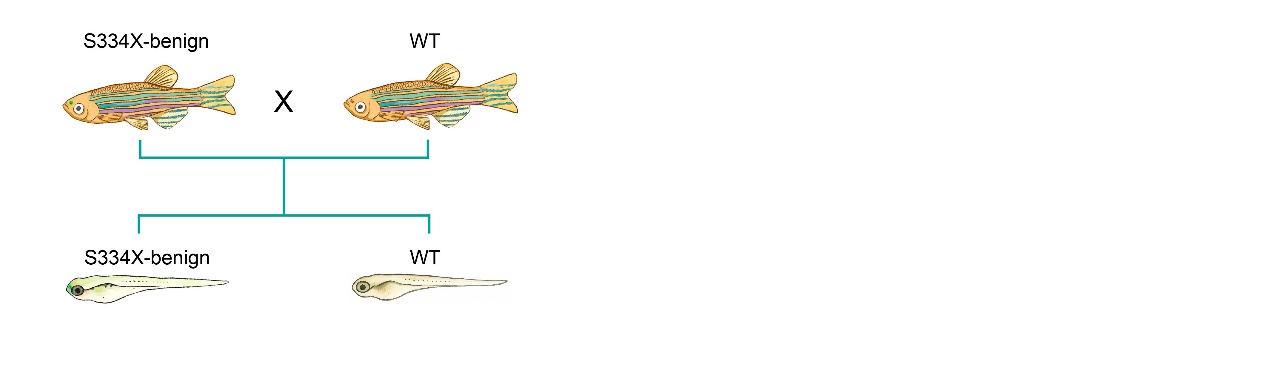


A schematic diagram of the breeding strategy for evaluating the heritability of benign phenotypes. The S334X-benign zebrafish adults were crossed with wild-type (WT) fish, and the resulting F3 offspring were genotyped using nose fluorescence (GFP). This strategy was used to assess whether the benign phenotype segregates according to Mendelian inheritance.

**Supplementary Figure 3 ATC insertion induces localized alterations in DNA shape features upstream of the S334X transgene.**


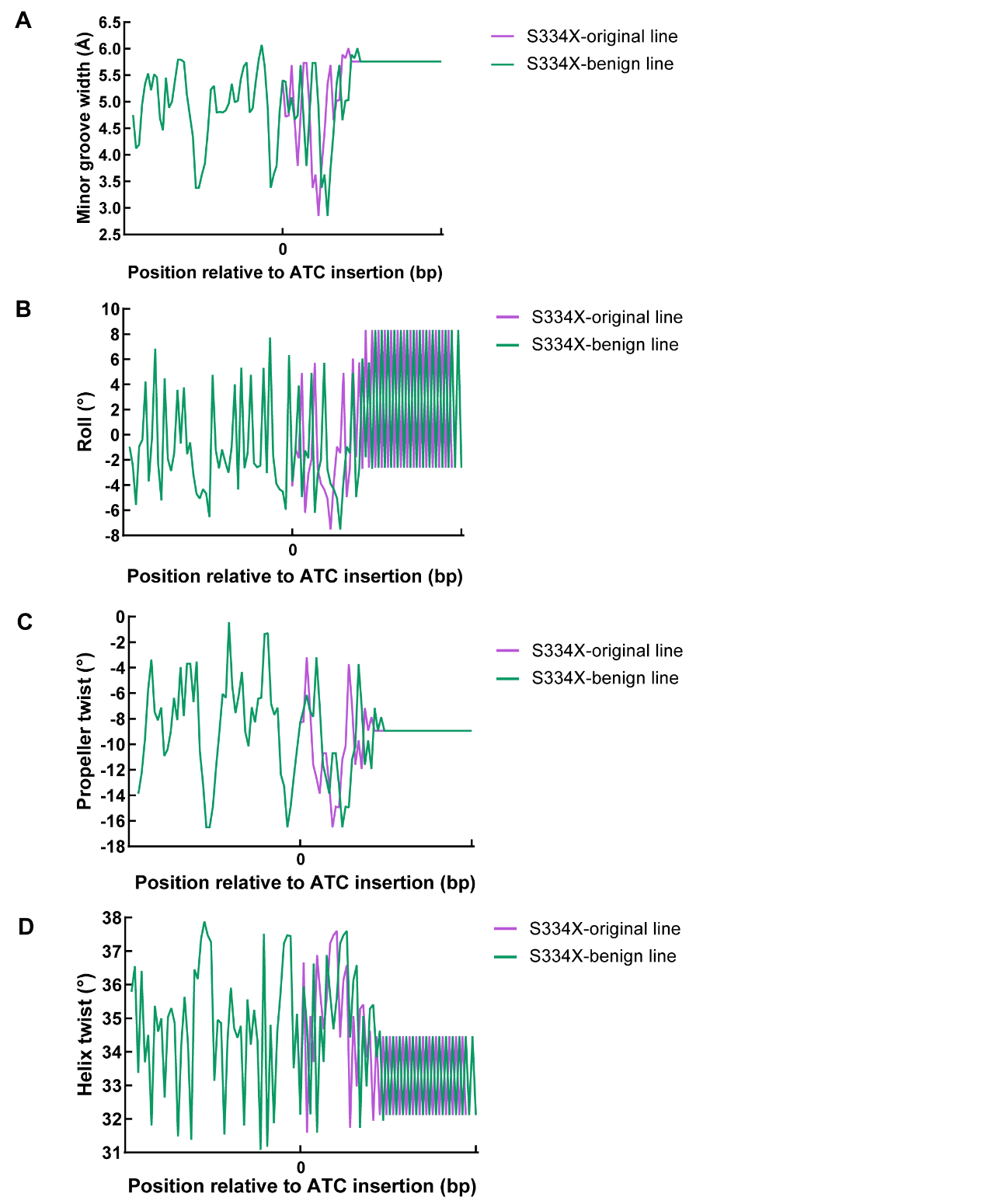


In silico DNA shape prediction was performed on genomic sequences flanking the ATC insertion site from the S334X-original and S334X-benign alleles using the DNAshape framework. **(A)** Minor groove width (MGW), **(B)** Roll, **(C)** Propeller twist (ProT), and **(D)** Helix twist (HelT) profiles are shown as a function of genomic position relative to the ATC insertion site (position 0). Upstream profiles were largely similar between the two alleles, whereas divergence became apparent at and downstream of the insertion site, reflecting altered local DNA shape predictions caused by the ATC insertion and its downstream sequence context.

**Supplementary Figure** **4**
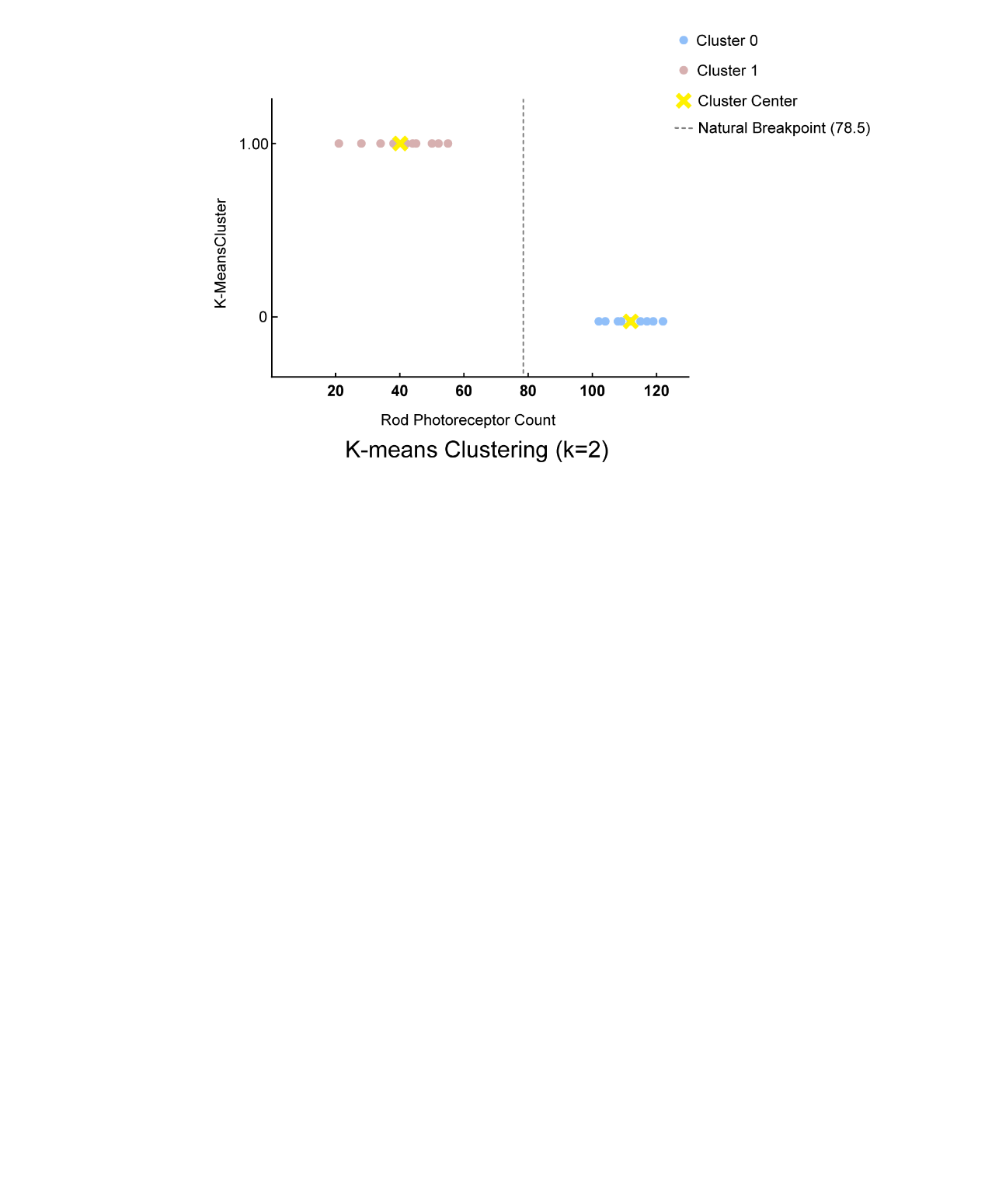


Rod photoreceptor counts of benign line at 15 dpf were subjected to K-means clustering with k = 2. Each dot represents an individual zebrafish, colored by cluster membership. Cluster centers are marked with yellow crosses (Cluster 0: 112; Cluster 1: 40.9). The vertical dashed line indicates the natural breakpoint at 78.5, previously determined by natural breakpoint analysis. The clustering results were consistent with the bimodal distribution revealed by natural breakpoint and KDE analysis, confirming the existence of two distinct phenotypic groups.

**Supplementary Figure** **5 Natural breakpoint analysis of rod photoreceptor counts in S334X-benign line individuals at different time points.**


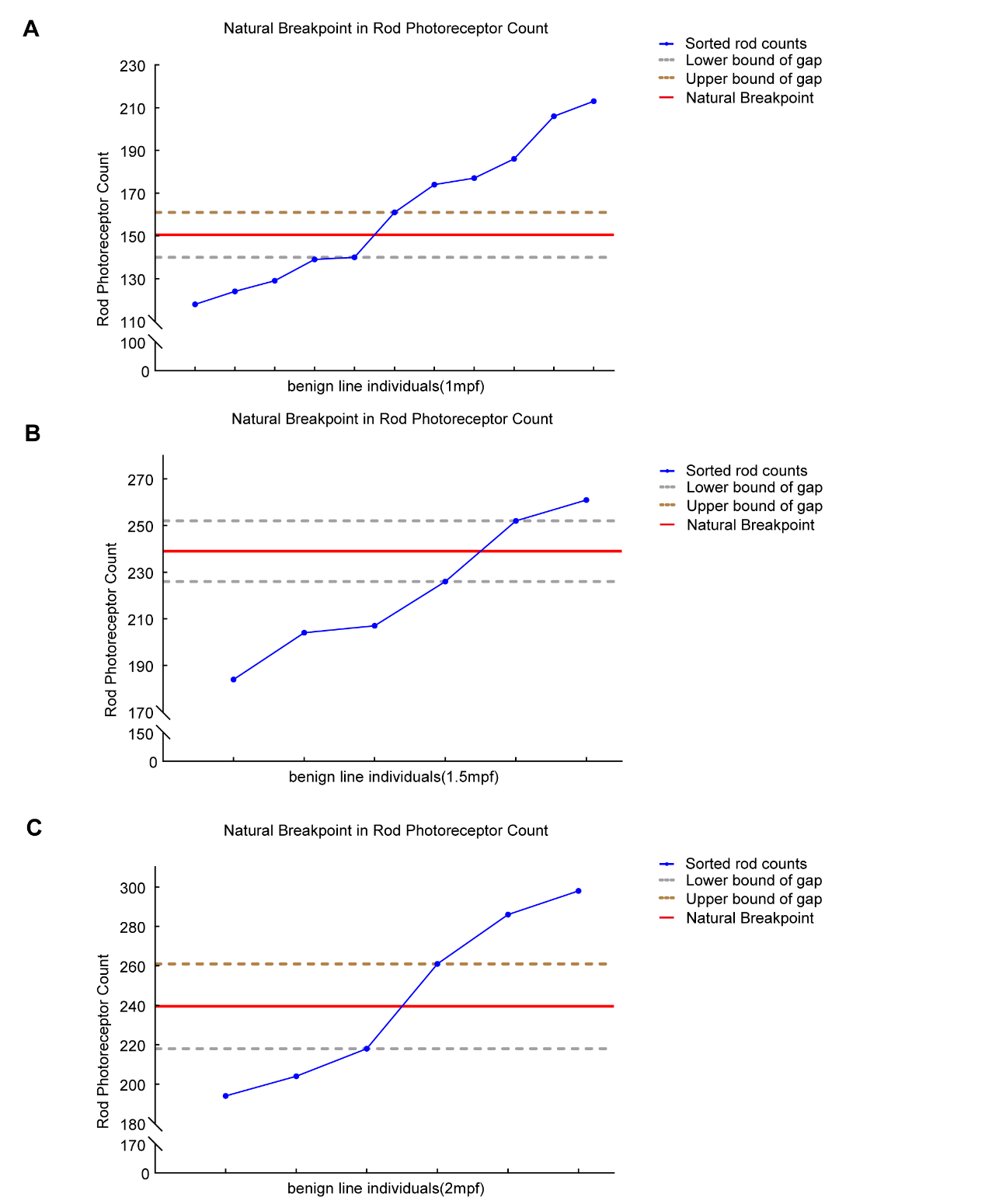


(A) At 1 mpf, rod counts revealed a natural gap between 140 and 161, yielding a calculated breakpoint at 150.5 (red line). (B) At 1.5 mpf, rod counts revealed a natural gap between 226 and 252, yielding a calculated breakpoint at 239 (red line). (C) At 2 mpf, rod counts revealed a natural gap between 218 and 261, yielding a calculated breakpoint at 239.5 (red line). In all plots, rod counts (Y-axis) are ranked and plotted against individual fish (X-axis), where each point represents a S334X-benign individual. The dashed lines indicate the lower (gray) and upper (brown) limits of the phenotypic gap, and the red lines indicate the selected breakpoint thresholds.

**Supplementary Figure** **6**


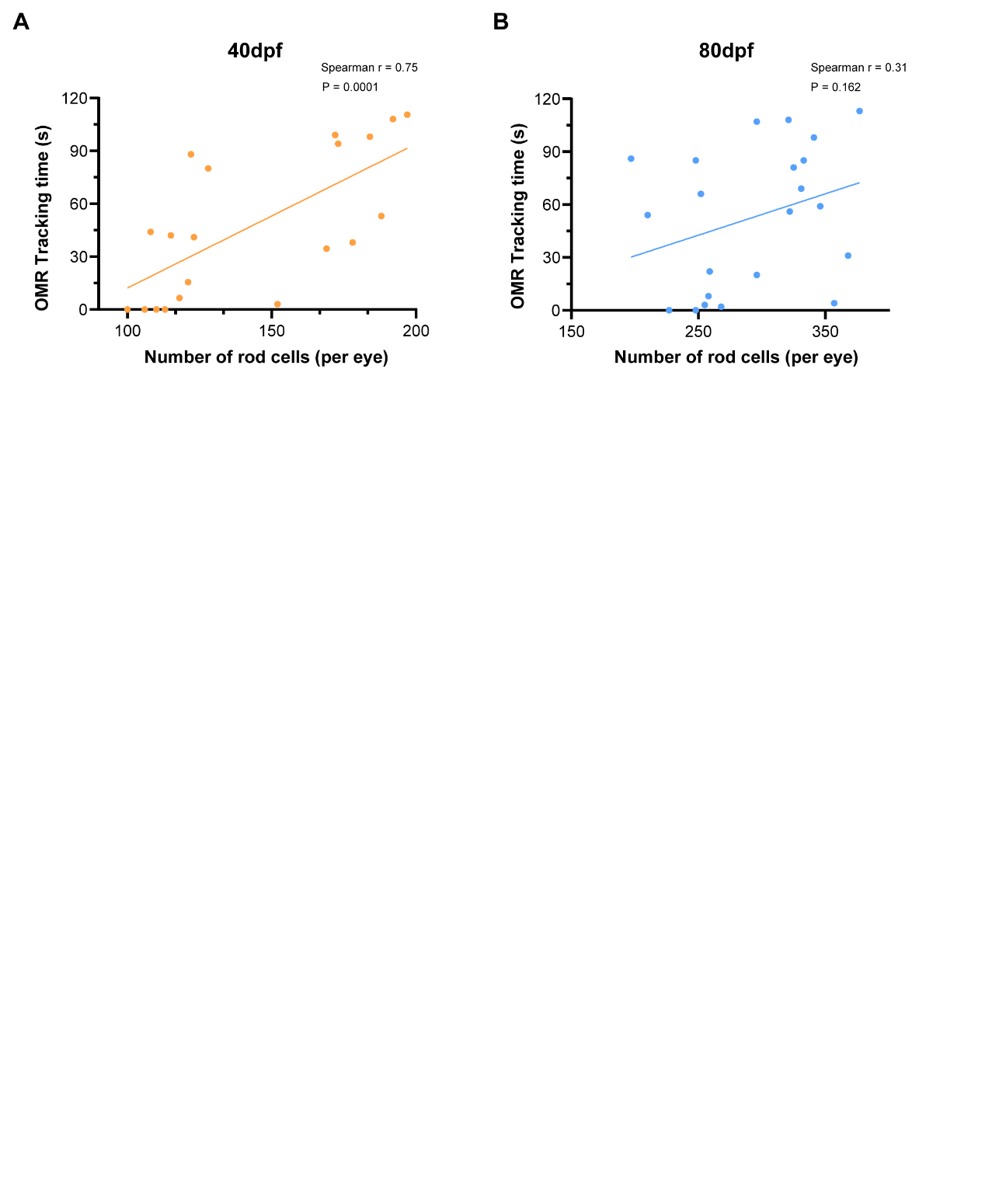


**(A)** At 40 days post-fertilization (dpf), a strong positive correlation was observed between the number of rod photoreceptors per eye and OMR tracking time. Each dot represents an individual fish. Spearman’s rank correlation analysis revealed a significant association (r = 0.75, P = 0.0001), indicating that visual behavior at this stage closely reflects rod photoreceptor preservation. **(B)** At 80 dpf, the relationship between rod photoreceptor number and OMR tracking time was markedly attenuated. Although a weak positive trend was observed, the correlation was not statistically significant (Spearman r = 0.31, P = 0.162).

**Supplementary Figure** **7**


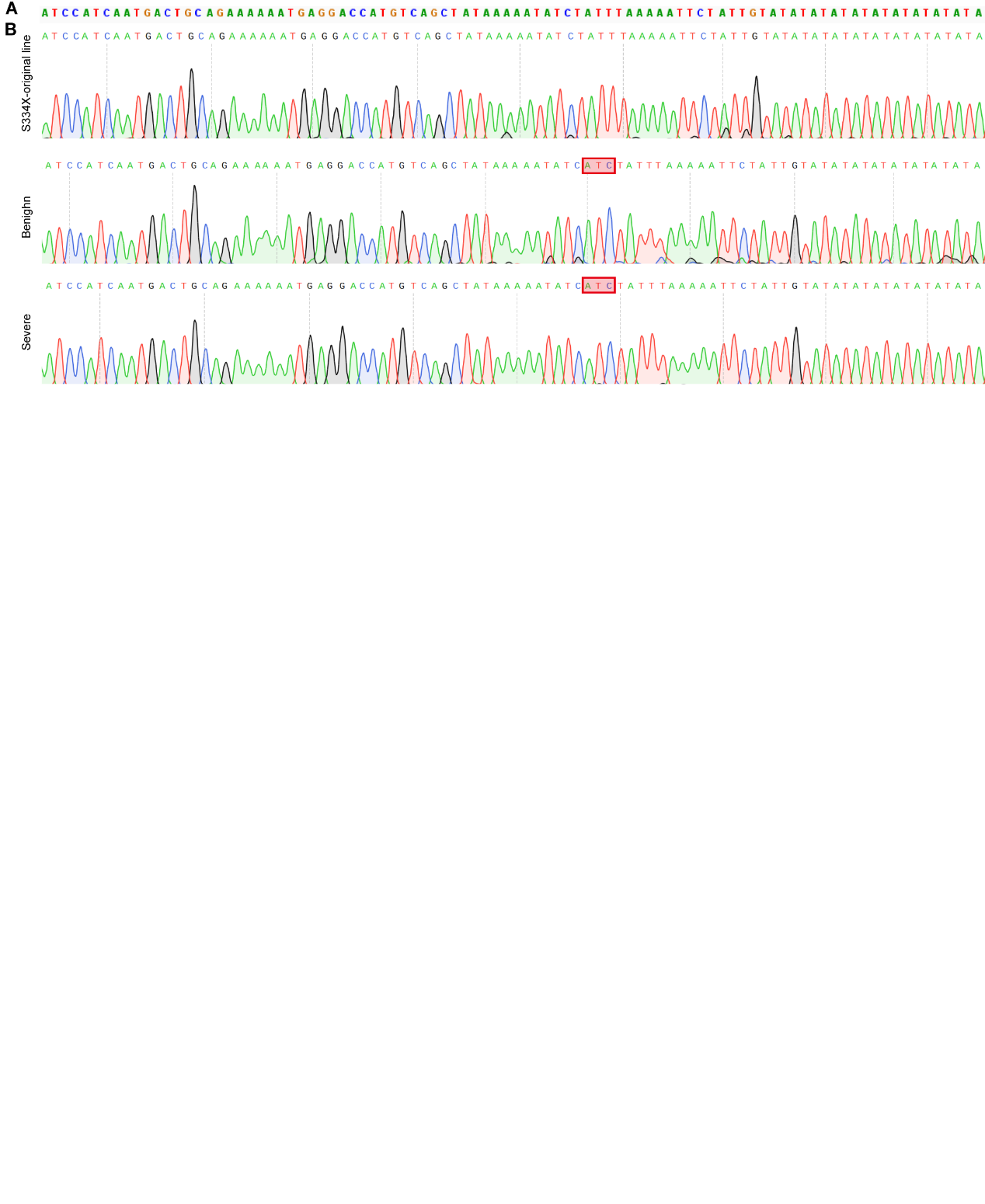


**(A)** Reference genomic sequence corresponding to approximately 2.4 kb upstream region of the transgene insertion site (chr10:41,004,588), demonstrating the original sequence context (as observed in the S334X-original line). **(B)** Cloning and sequencing of genomic DNA from the three lines confirms the 3 bp insertion at chr10:41,004,588 in both benign and severe phenotypes of S334X-benign line (middle and bottom panel). The corresponding region in the S334X-original (F3) line lacks the insertion (top panel)

**Supplementary Figure** **8**

(A) Schematic diagram of the Tol2-based transgenic construct pTol2-*rh1*-hsRHO-OmpEGFP used to generate zebrafish expressing the human RHO S334X mutant.

(B) Schematic representation of the breeding strategy from F0 to F1 generations of zTg(*rh1*:hsRHO:Omp:EGFP) zebrafish. Rod photoreceptor visualization was achieved by crossing with *rh1*: EGFP fish and the transgenic individuals were isolated under fluorescence microscopy at 5 dpf.


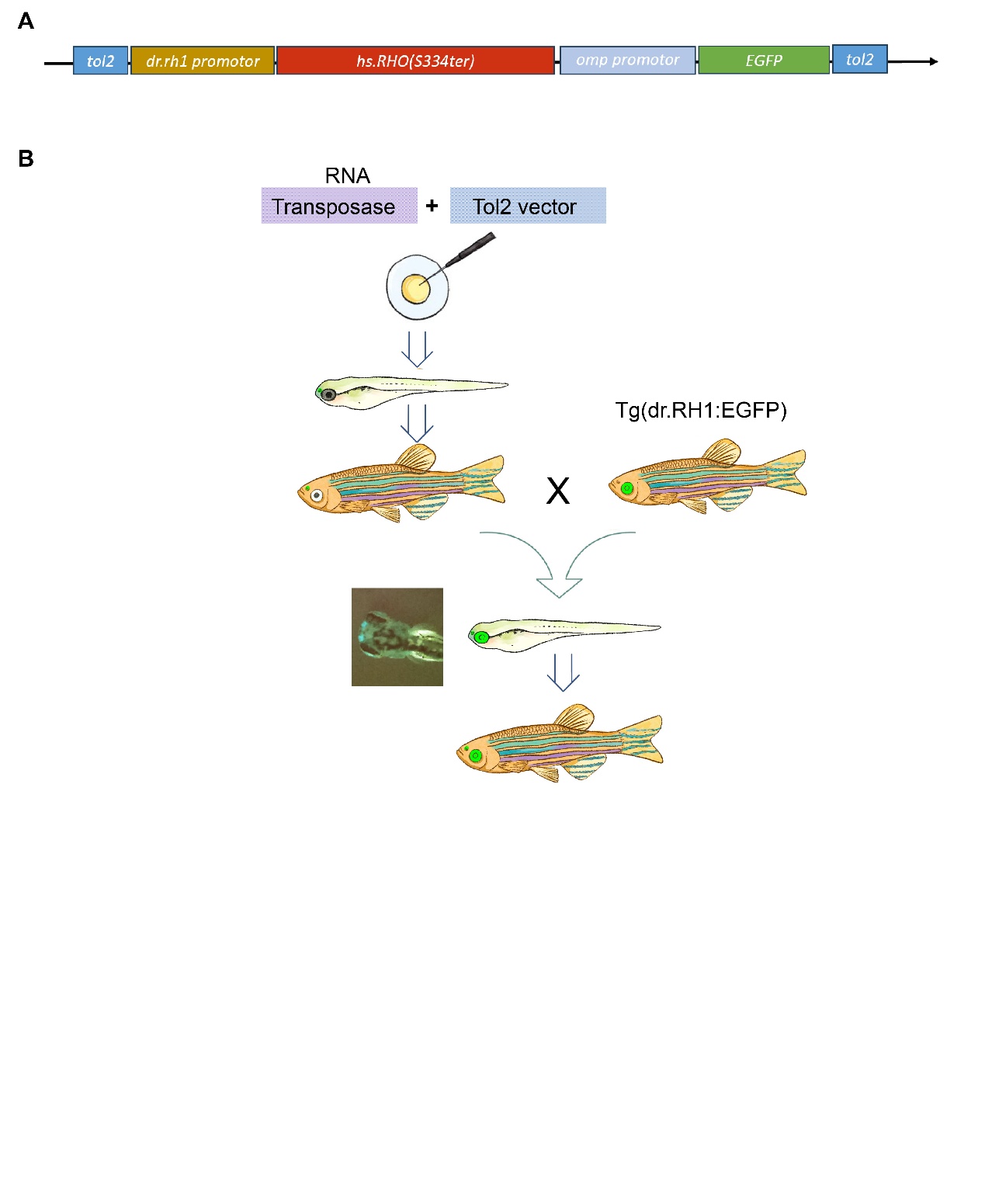


(A) Schematic diagram of the Tol2-based transgenic construct pTol2- *rh1*-hsRHO-OmpEGFP used to generate zebrafish expressing the human RHO S334X mutant.

(B) Schematic representation of the breeding strategy from F0 to F1 generations of zTg(*rh1*:hsRHO:Omp:EGFP) zebrafish. Rod photoreceptor visualization was achieved by crossing with *rh1*: EGFP fish and the transgenic individuals were isolated under fluorescence microscopy at 5 dpf.

**Supplementary Table 1.** **Summary of predicted transcription factor motif families surrounding the ATC insertion site**

| **Motif family** | **Representative TF** | **Motif cluster region (bp)** | **Benign** | **original** |
| --- | --- | --- | --- | --- |
| FOX | FOXL1 | 77-108 | Yes | Yes |
| GATA | Gata1 | 6-11 | Yes | Yes |
| bZIP family (MAF/NRF) | MAFG::NRF1 | 11-16 | Yes | Yes |
| TBP/TATA-box binding | TBP | 44-50 | Yes | Yes |

**Supplementary Table 2.** **PCR primers for mutation site confirmation and insertion site confirmation.**

| **Number** | **Gene ID** | **Forward (5'-3')** | **Reverse (5'-3')** | **Amplicon size (bp)** |
| --- | --- | --- | --- | --- |
| 1 | RHO | GCGTGGCATTCTACATCTTC | GTGGTATGGCTGATTATGATC | 318 |
| 2 | Danio rerio genome  (Chromosome 10) | TCCTTTGAGGTGGTGGTTCG | CCTGCGCTTCTCTGTCATGT | 1197 |
| 3 | Danio rerio genome  (Chromosome 10) | CACATGGTCCTGCTGGAGTT | CACTTGAACGAAAAGTCCTGGT | 1105 |

The qPCR primers are shown in the table, No.1: The forward primer was derived from the RHO sequence, the reverse primer was designed from the vector, and the product contained the mutation site; Number 2: forward primers (genomic sequence upstream of the insertion site) and reverse primers (sequence within the vector) designed for the insertion site (zebrafish chromosome 10),the size of the product was 1197bp;Number 3: forward primers (in-vector sequences) and reverse primers (genomic sequences downstream of the insertion site) designed for the insertion site (zebrafish chromosome 10),the size of the product was 1105bp.

**Supplementary Table 3. qPCR primer sequences.**

| **Number** | **Gene ID** | **Forward (5'-3')** | **Reverse (5'-3')** | **Amplicon size (bp)** |
| --- | --- | --- | --- | --- |
| 1 | RHO | GCGTGGCATTCTACATCTTC | GTGGTATGGCTGATTATGATC | 318 |
| 2 | Gapdh | GCAGTCTTTTGGAAGATTTGCCT | ATGGGAGAATGGTCGCGTAT | 350 |
| 3 | Actb1 | TTCAGTGCACGCTGAGAAGA | CCAACCATCACTCCCTGATGT | 199 |

The qPCR primers listed in the table were used to quantify gene expression.
Gapdh and Actb1 served as internal reference genes for normalization.

- **No. 1**: RHO-specific primers targeting the mutation site.
- **No. 2**: Gapdh primers for reference normalization.
- **No. 3**: Actb1 primers for reference normalization.
